# Dynamic Alternative Splicing During Mouse Preimplantation Embryo Development

**DOI:** 10.3389/fbioe.2020.00035

**Published:** 2020-02-07

**Authors:** Yongqiang Xing, Wuritu Yang, Guoqing Liu, Xiangjun Cui, Hu Meng, Hongyu Zhao, Xiujuan Zhao, Jun Li, Zhe Liu, Michael Q. Zhang, Lu Cai

**Affiliations:** ^1^School of Life Science and Technology, Inner Mongolia University of Science and Technology, Baotou, China; ^2^The Inner Mongolia Key Laboratory of Functional Genome Bioinformatics, Inner Mongolia University of Science and Technology, Baotou, China; ^3^The Key Laboratory of Mammalian Reproductive Biology and Biotechnology of the Ministry of Education, Inner Mongolia University, Hohhot, China; ^4^Department of Biological Sciences, Center for Systems Biology, The University of Texas at Dallas, Richardson, TX, United States

**Keywords:** alternative splicing, gene expression, preimplantation development, zygotic gene activation, splicing factors

## Abstract

The mechanism of alternative pre-mRNA splicing (AS) during preimplantation development is largely unknown. In order to capture the dynamic changes of AS occurring during embryogenesis, we carried out bioinformatics analysis based on scRNA-seq data over the time-course preimplantation development in mouse. We detected numerous previously-unreported differentially expressed genes at specific developmental stages and investigated the nature of AS at both minor and major zygotic genome activation (ZGA). The AS and differential AS atlas over preimplantation development were established. The differentially alternatively spliced genes (DASGs) are likely to be key splicing factors (SFs) during preimplantation development. We also demonstrated that there is a regulatory cascade of AS events in which some key SFs are regulated by differentially AS of their own gene transcripts. Moreover, 212 isoform switches (ISs) during preimplantation development were detected, which may be critical for decoding the mechanism of early embryogenesis. Importantly, we uncovered that zygotic AS activation (ZASA) is in conformity with ZGA and revealed that AS is coupled with transcription during preimplantation development. Our results may provide a deeper insight into the regulation of early embryogenesis.

## Introduction

Decoding molecular mechanisms of totipotency and pluripotency is crucial to the understanding of reproductive biology and to regenerative medicine (Hamatani et al., 2004). Preimplantation process, which encompasses the period from fertilization to implantation, is a fundamental developmental stage that has been extensively studied in order to gain insight to totipotency and pluripotency (Yan et al., 2013; Petropoulos et al., 2016). With the development of single-cell RNA-seq (scRNA-seq) technology, the barrier of scarcity of preimplantation embryo materials has been overcome. The scRNA-seq is an unbiased and popular approach to investigate heterogeneous tissues and organs, especially for embryogenesis. To date, numerous scRNA-seq studies on mouse or human preimplantation embryos have identified a large number of genes and signaling pathways involved in early stages of embryonic development (Hamatani et al., 2004, 2006; Yan et al., 2013; Petropoulos et al., 2016). However, the molecular regulatory mechanisms underlying preimplantation process remain incompletely understood, especially the effect of AS in this process.

AS is a ubiquitous and conserved regulatory mechanism of gene expression in which introns are removed and exons are joined in different combinations to create various alternative mRNA products (Zhang, 2002; Park et al., 2018). The distinct proteins produced from identical pre-mRNAs via AS may have different, even antagonistic functions (Park et al., 2018). AS greatly expands the diversity of transcriptome and proteome in higher eukaryotic organisms and plays an important role in numerous processes, such as cell differentiation, proliferation, apoptosis, organ development and the genesis of human disease, etc. (Kornblihtt et al., 2009; Kalsotra and Cooper, 2011; Singh and Cooper, 2012; Xiong et al., 2015; Scotti and Swanson, 2016). AS is also essential for mammalian early embryogenesis to generate a viable organism from a fertilized cell (Revil et al., 2010). Revil et al. (2010) studied splicing-sensitive exon microarray in embryonic 8–12 days mouse embryos and revealed that AS is frequent across early developmental stages and tissues. However, the detailed temporal and spatial patterns of AS during preimplantation development are poorly understood.

In mouse, pre- and early embryo development is a complex process that consists of sequential maturation events of the oocyte, fertilization (zygote) and embryo growth (2-cell, 4-cell, 8-cell, morula, and blastocyst) (Assou et al., 2011). Here, we utilized time-series scRNA-seq data consisting 21 single-cells from mouse seven consecutive stages of preimplantation development to dissect the dynamics of the gene expression and AS. A total of 4,952 genes were differentially expressed at the gene level (DEGs) in all the consecutive early developmental stages, of which 507 genes were also differentially alternatively spliced. The AS atlas was constructed for seven development stages and 1,170 differential AS events (DAS) in 836 genes were identified at the consecutive development stages. A regulatory cascade of AS that some splicing factors regulate AS by DEGs and DAS of their own gene transcript was found. A dataset of ISs during preimplantation development was established. Moreover, we uncovered that ZASA is in conformity with ZGA and revealed that AS is coupled with transcription during preimplantation in mouse. This study is expected to be helpful for elucidating the molecular and cellular mechanisms of preimplantation embryo development.

## Materials and Methods

### Dataset

Fan et al. developed SUPeR-seq (single-cell universal poly(A)-independent RNA sequencing) method to sequence single cell complete transcriptome (poly(A)+ and poly(A)–) of mouse early embryos (Fan et al., 2015). We downloaded complete transcriptome data of 25 single cells generated from mouse occytes and preimplantation embryos. The embryos cover seven consecutive stages of preimplantation development: metaphase II oocyte, zygote, 2-cell, 4-cell, 8-cell, morula, and blastocyst. Then, we respectively dropped two poor-quality single-cells transcriptome data in occyte and zygote, RNA-seq data at every development stage of mouse preimplatation was composed of three single-cell sequenced on Illumina HiSeq 2,000 platform (Table S1). On average, every cell has 12.7 million in 101 bp paired-end reads.

FastQC v0.11.8 (Andrews, 2010) and Trimmomatic v.38 (Bolger et al., 2014) were used to perform QC (quality control) analysis for raw reads. FastQC analysis showed the adaptor was already cut before uploading GEO and quality of 3′ end of reads is lower. We removed low quality reads (the average quality per base within 4-base wide window drops below 10, SLIDINGWINDOW:4:10). The reads containing poly(A)24/(T)24 sequences were trimmed off. The leading and trailing bases of a read were cut if quality is below 3 (LEADING:5, TRALING:5). All reads were outputted with read length of 91 bp (MINLEN:91, CROP:91). The average surviving rate and sequencing depth of paired-end reads after quality control is 79.1% and 10.0 million (Table S1).

### Quantification of Transcript and Gene Expression

The gene annotation GTF file, nucleotide sequence FASTA file and transcript sequence FASTA file were downloaded from Gencode (vM10/GRCm38.p4). In this work, we only focused on coding gene. After filtering, the annotation GTF file composed of 22,021 coding genes was created.

The transcript qualification of different preimplantation development stages was carried out by combing Salmon v0.11.3 (Patro et al., 2017) and transcript sequence FASTA file. For indexing, because the read length is larger than 75 bp, we used the quasi mapping mode to build an auxiliary *k*-mer hash over *k*-mers of length 31 (-type quasi -k 31). Besides, the option to qualify duplicate transcripts (“-keepDuplicates”) was turned on. For accurate quantification, the option to correct for the sequence specific bias (“-seqBias”) was also turned on and all other parameters were on default settings. The TPM (Transcripts Per Kilobase Million) value of 86,623 transcripts corresponding all coding genes across all samples was calculated (Table S2).

To construct gene count matrix (22,021 × 21), TPM data of transcript generated by Salmon was processed using tximport version 1.10.1 R package (Soneson et al., 2015) with the default setting (Table S3).

### The Identification of Differentially Expressed Genes

Gene expression analysis and cell type clustering were performed using Seurat v2.3.4 (Butler et al., 2018). Seurat is an R package designed for QC analysis, visualization, and exploration of single cell RNA-seq data. Seurat aims to enable users to identify and interpret sources of heterogeneity from single cell transcriptomic measurements, and to integrate diverse types of single cell data. By QC, only those genes that were expressed in at least 3 or more cells and cells that expressed more than 10,000 genes were retained. A 16,539 (genes) × 21 (samples) Seurat object was created. After removing low-expressed genes, the “LogNormalize method” was used to normalize the gene expression. Next, the FindVariableGenes function was used to identify highly variable genes followed by scaling data (ScaleData) for downstream analysis. We clustered the cells using FindClusters function and visualized all cells by integrated tSNE. Finally, we used FindMarkers() function of Seurat to detect differentially expressed genes under every consecutive stages of preimplantation development. FindMarkers() function provides nine tests for differential expression which can be set with the test.use parameter. Here, test.use was set to DESeq2, which is based on a model using the negative binomial distribution (Love et al., 2014). The *avg_log*_*e*_ *fold change* (*FC*) of gene abundances was calculated in each consecutive development stages. *P*-values were adjusted by the BH method for multiple testing correction (Benjamini and Hochberg, 1995). We selected adjust *p* value ≤ 0.05 and |*avg*_log_*e*_*FC*| ≤ 0.25 as the threshold to judge the significance of differentially expressed genes.

### The Identification and Quantification of AS Events

At present, there are many tools to detect and quantify AS events, such as SUPPA (Trincado et al., 2018), rMATs (Shen et al., 2014), MAJIQ (Vaquero-Garcia et al., 2016), etc. On the one hand, SUPPA is much faster than the other methods and achieves higher accuracy compared to other methods, especially at low sequencing depth and short read length (Trincado et al., 2018). On the other hand, this work only paid attention on AS events derived from pre-existing transcript annotations. Thus, AS analysis in this work was performed by SUPPA v2.3 (Trincado et al., 2018). SUPPA is a powerful and reliable tool to study splicing at the transcript isoform or at the local AS event level across multiple conditions. SUPPA was used to generate the AS events (e.g., A5SS, A3SS, SE, RI, MXE, AFE, ALE) from mouse annotation file. Then, AS event inclusion levels (*PSI*) from multiple developmental stages were quantified. Furthermore, SUPPA calculated the magnitude of splicing change (Δ*PSI*) and its significance across multiple development stages directly from TPM value of transcript involved in the event. For example, an exon skipping event across two development stages consists of an included transcript and a skipped transcript. Then, the included level *PSI* and splicing change Δ*PSI* can be defined as:

$$\begin{array}{l} \;PSI=\frac{TPM_{1}}{TPM_{1}+TPM_{2}}\\ \Delta PSI=\bar{PSI_{1}}-\bar{PSI_{2}} \end{array}$$

where the *TPM*_1_ and *TPM*_2_ are the expression level of included transcript and skipped transcript, respectively. $\bar{PSI_{1}}$ and $\bar{PSI_{2}}$ are the mean of *PSI* of biological replicates for development stage 1 and development stage 2, respectively.

Criteria for judging DAS was that in contrast group (1) splicing change (Δ*PSI*) across two different developmental stages showed ≥ 0.1. (2) Δ*PSI* differs significantly with *p* value ≤ 0.05 (Calixto et al., 2018).

### Identification of ISs

For the isoform switch analysis, we used the TSIS R package, which is a tool to detect significant transcript ISs in time-series data (Guo et al., 2017). ISs between any two consecutive development stages were identified using the default parameters in which (1) the probability of switch (i.e., the frequency of samples reversing their relative abundance at the switches) was set to >0.5; (2) the sum of the average differences of the two isoforms in both intervals before and after the switch point were set at ΔTPM >1; (3) the significance of the differences between the switched isoform abundances before and after the switch was set to *p* < 0.05; and (4) both intervals before and after switch must consist of at least 2 consecutive development stages to detect long lasting switches.

### Gene Ontology and KEGG Enrichment Analysis

Gene Ontology and KEGG pathway enrichment analysis were performed using clusterProfiler package in R (http://bioconductor.org/packages/release/bioc/html/clusterProfiler.html) (Yu et al., 2012). The statistical significance threshold level for all GO enrichment and KEGG pathway analyses was *p.adjust* < 0.05.

### Splicing Factor Analysis

A total of 446 mouse splicing factors were selected for analysis based on literature mining for previously described splicing functions (Han et al., 2013; Goldstein et al., 2017), and “RNA splicing” or “spliceosome”-associated Gene Ontology (GO) terms from MGI (Smith et al., 2018) (http://www.informatics.jax.org/marker) (Table S4).

## Results

### The Global Outlook of DEGs and DAS During Preimplantation Development

To examine changes in gene expression and AS of mouse preimplantation embryos, we collected complete transcriptome data of 21 single cells from mouse occytes and preimplantation embryos (Fan et al., 2015). The embryos cover seven consecutive stages of preimplantation development: metaphase II oocyte, zygote, 2-cell, 4-cell, 8-cell, morula and blastocyst (see Materials and Methods**)**. Every development stage includes three scRNA-seq replicates sequenced on Illumina HiSeq 2000 platform. On average, every cell has 12.7 million in 101 bp paired-end reads (Table S1). QC analysis of RNA-seq data was carried out using FastQC (Andrews, 2010) and Trimmomatic (Bolger et al., 2014). Consequently, the average clean paired-end reads in every cell is ~10.0 million with length of 91 bp (Table S1).

**Figure 1:**
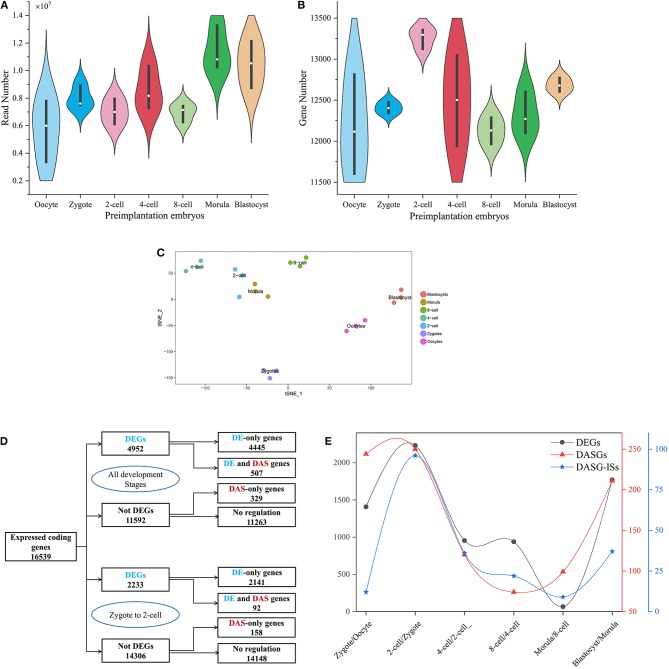
A schematic description of dataset, DEGs and DAS. **(A,B)** is the frequency distribution of reads mapped to protein-coding genes and protein-coding genes captured at every preimplantation development stage. The white circle denotes median of all values. Black rectangle denotes interquartile range (quartile to third quartile). **(C)** Cell type assignment using the most variable genes across all preimplatation development stages following t-SNE-based visualization of 21 cells. Cells that marked with same color were clustered at the same developmental stage. **(D)** The number of DEG and DASGs at all preimplantation development stages. The blue denotes DEGs and the red denotes DASGs. **(E)** The number of DEGs, DASGs and DASG-ISs for every consecutive stages of preimplantation development. DASGs represent genes in which DAS was identified. DAS-ISs represent isoform switches identified from DASGs.

In this study, we only focused on coding genes in mouse annotation GTF file. After filtering, 22,021 coding genes were considered in the downstream analysis. We first examined how many reads mapped to coding genes in every cell (Figure 1A). On average, every cell includes ~8.3 million reads. Then, Salmon tool (Patro et al., 2017) and tximport R package (Soneson et al., 2015) were employed to quantify expression matrix of transcripts and genes. Across all samples, we identified 18,272 protein-coding genes expressed in at least one sample. In total, 6,522 protein-coding genes were expressed in all samples. We selected the protein-coding genes that were expressed in at least 3 or more cells for downstream analysis. With this criterion, the gene count matrix was created, which includes 16,539 protein-coding genes along the rows and 21 samples along the columns. We observed that ~82% protein-coding genes were expressed during preimplantation development. The mean of detected protein-coding genes is 12,496 across 21 cells and the number of detected protein-coding genes in every cell is higher than 10,000 (Figure 1B). The mean of detected protein-coding genes with TPM larger than 10 across 21 cells is 5,692 (Table S1). The number of protein-coding genes (1,3259) in the 2-cell stage is larger than other developmental stages. ZGA is the first major developmental event that occurs following fertilization (Schultz et al., 2018). After ZGA process, the genetic program governed by maternal transcripts/proteins should be switched to that dominated by transcripts/proteins from the newly formed zygotic genome (Kanka, 2003; Hamatani et al., 2004). During ZGA process of mouse embryos development, lots of zygotic genes are activated and maternal genes have not been degraded thoroughly. Given that 2-cell stage is major start of ZGA in mouse (Abe et al., 2018), the fact that the maximum number of protein-coding genes was observed in the 2-cell stage is reasonable.

The global gene expression profiles at different developmental stages should be distinguishable. Seurat is a widely used R package for scRNA-seq data analysis (Butler et al., 2018). Especially, Seurat was often used to identify cell identity. We applied Seurat to 16,539 (genes) × 21 (samples) count matrix in mouse embryos development. After removing low-expressed genes and normalizing the gene expression, 6,902 highly variable genes throughout each development stage were identified. In order to confirm the identity of every cell, we applied a graph-based clustering method on the most variable genes and identified seven clusters of cells as visualized by bi-dimensional t-distributed stochastic neighbor embedding (t-SNE) (Figure 1C). By comparing with the experimental source of cells, we observed that every cell can be clustered correctly as actual development stages. Thus, 21 cells selected as samples have a high reliability for downstream analysis.

Developmental process requires precise spatial-temporal regulation of gene expression and AS. Here, we used time-series scRNA-seq to examine the dynamics of DEGs at the gene level and DAS at transcript level. To analyze the time-series RNA-seq data at gene levels, we employed FindMarkers() function of Seurat package to detect DEGs between each two consecutive stages of the preimplantation development. If *avg_log*_*e*_*FC* is positive values, it indicates that the gene is more expressed in the first group, and vice versa. Here, we used a more stringent criteria: a gene was regarded as differentially expressed if |avg_log_e_FC| ≥ 0.25 (≥1.3-FC) and adjusted *p* value ≤ 0.05. Under these criteria, a total of 4,952 genes were identified as differentially expressed throughout all successive preimplantation developmental stages (Figure 1D). Of these, 37.5% were consistently up-regulated, 35.5% were consistently down-regulated and 27.0% were up-regulated or down-regulated over different consecutive development stages. Moreover, we applied SUPPA tool on the transcript-level data generated by Salmon (Trincado et al., 2018) to identify genes that were DAS between consecutive preimplantation developmental stages. We recognized 836 DASGs, of which 507 are overlapped with DEGs and 329 are not. It indicates that 507 genes are simultaneously regulated at both transcriptional level and AS level, and 329 genes are only regulated by AS. As a typical example, a total of 2,233 DEGs and 250 DASGs between zygote and 2-cell were detected. Of DASGs, 92 are also DEGs. Furthermore, heatmap showing the expression levels of DEGs (Figure S1) and the inclusion levels of DAS events (Figure S2) across seven consecutive stages of preimplantation development suggested some DEGs and DAS events is stage-specific. So, the DEGs and DAS events were analyzed in detail.

#### Analysis of DEGs in Consecutive Developmental Stages of Preimplantation Embryo

**Figure 2:**
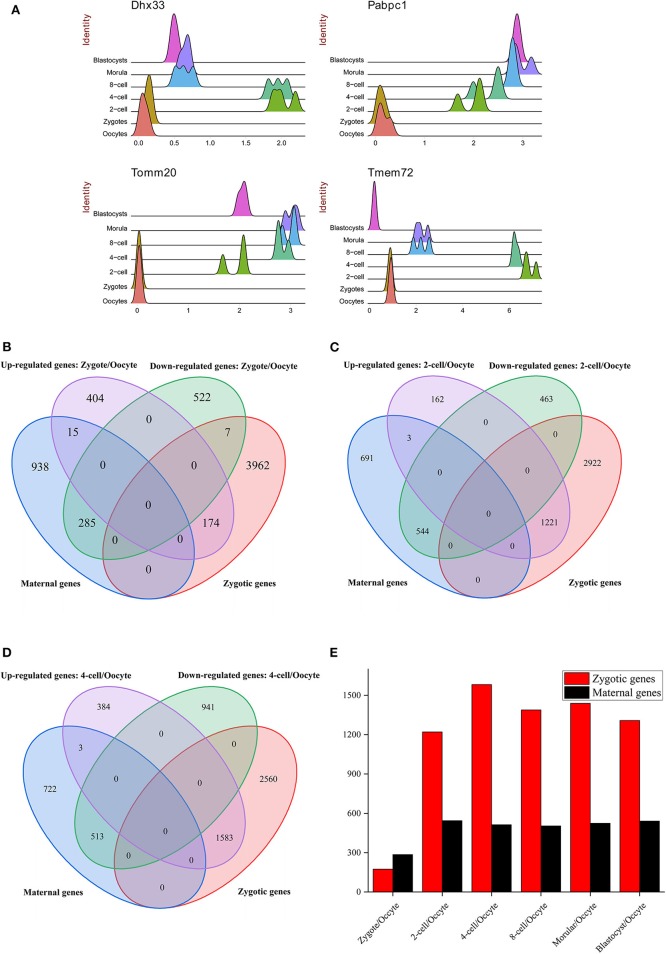
The distribution of DE genes. **(A)** The expression atlas of significantly up-regulated genes in different preimplantation development stages. The gene expression level was normalized by Seurat (see Materials and Methods). **(B–D)** The intersection between DE genes (**B**: zygote/oocyte, **C**: 2-cell/oocyte, **D**: 4-cell/oocyte) and maternal/zygotic genes. The maternal and zygotic gene sets were derived from Fan et al. (2015). **(E)** The distribution of DE genes overlapped with maternal and zygotic genes. Zygotic genes marked with red color denotes the up-regulated genes overlapped with zygotic genes from Fan et al. (2015). Maternal genes marked with black color denotes the down-regulated genes overlapped with maternal genes from Fan et al. (2015).

ZGA is essential for replacing the degraded maternal transcripts with zygotic transcripts (Yan et al., 2013). In mouse embryos, major ZGA process reportedly occurs at the 2-cell and 4-cell stages (Abe et al., 2018). The greatest DEGs number between 2-cell and zygote compared with other consecutive stages during preimplantation development was detected (Figure 1E). It indicates that the transcriptome difference between these two stages is greatest. By functional enrichment analysis on DEGs, we confirmed some previous conclusions, such as the zygotic-specific transcription and translation machinery is established during ZGA (Figures S3, S4 and Table S5) (Yan et al., 2013). Besides, some genes were also strongly enriched in splicing-associated processes, such as mRNA processing (gene number = 61, *p.adjust* = 1.19 × 10^−12^), RNA splicing (gene number = 50, *p.adjust* = 3.44 × 10^−09^), mRNA catabolic process (gene number = 38, *p.adjust* = 2.62 × 10^−08^), and alternative mRNA splicing (gene number = 10, *p.adjust* = 0.047), indicating that the biological process of mRNA splicing are activated in ZGA (Table S5). The significantly up-regulated gene *Dhx33* in 2-cell stage plays essential roles in mRNA translation, pre-mRNA splicing and ribosome biogenesis (Zhang et al., 2015) (Figure 2A). The pabpc1 protein that binds the poly(A) tail of mRNA involved in cytoplasmic regulatory processes of mRNA metabolism, such as pre-mRNA splicing. We found the expression level of gene *pabpc1* is significantly up-regulated in 2-cell (Figure 2A). This finding implied that AS may initiate in ZGA. It was known that mitochondrial metabolism contributes a major role in the supply of ATP during preimplantation embryo development (Wilding et al., 2009). The *Tomm20* gene is critical for synthesis of mitochondrial pre-proteins. The substantial amounts of ATP are consumed during ZGA. Thus, the expression level of *Tomm20* gene is elevated obviously during ZGA (Figure 2A). The most up-regulated gene in the 2-cell stage is *Tmem72* [Transmembrane protein 72-like, FC (2-cell/Zygote) = 403]. *Tmem72* encodes a transmembrane protein and the biological function of *Tmem72* is unknown. *Tmem72* is localized to the mitochondria in human clear cell renal cell carcinoma and is associated with metastasis (Wrzesinski et al., 2015). Further research is required to investigate the reason for the up-regulation of *Tmem72* in 2-cell and 4-cell stage compared to other development stages and its functional role in the mouse preimplantation development (Figure 2A). We also analyzed the expression profiles of occyte-specific genes including *Oas1e, Aspm, Rgs2, Fbxw28*, etc. (Figure S5).

In mouse preimplantation development, zygotic genes are activated until 4-cell stage. A total of 1,770 up-regulated genes and 1,428 down-regulated genes between 4-cell and zygote stages were identified. GO enrichment analysis results for these DEGs were similar with DEGs between 2-cell and zygotic stages (Figure S6, Table S6). Besides, the DEGs number between 8-cell and morular is smallest compared with other consecutive stages of preimplantation development, suggesting that the transcriptomes of these two stages of embryos are similar.

By using SUPeR-seq, Fan et al. (2015) identified 1,238 annotated maternal genes and 4,143 annotated zygotic genes. In order to illuminate ZGA process, DEGs before and after ZGA were compared with maternal and zygotic genes. Obviously, compared with oocyte, up-regulated and down-regulated genes in zygote, 2-cell and 4-cell are overwhelmingly overlapped with zygotic and maternal genes, respectively (Figures 2B–D). By comparing the overlapped genes under different development stages, it was suggested that ZGA initiates during one-cell stage, bursts during 2-cell stage and hit the peak during 4-cell stage (Figure 2E). This conclusion is consistent with Abe et al. (2018). They concluded that ZGA in mouse initiates at the mid-one-cell stage (minor ZGA) and is dramatically activated after 2-cell stage (major ZGA). If minor ZGA was inhibited transiently, most of embryos were arrested at the 2-cell stage. Thus, minor ZGA is crucial for the maternal-to-zygotic transition (Abe et al., 2018).

#### DAS Profiles in Consecutive Developmental Stages of Preimplantation Embryo

It has been well-known that pre-mRNA splicing can occur co-transcriptionally on nascent transcripts. However, isoforms abundance generated by AS may be masked by gene-level measurement. Here, we posed two questions: (i) when is pre-mRNA splicing activated? (ii) is AS activation coupled with ZGA? To answer these questions, we systematically examined the dynamics of AS during mouse preimplantation embryonic development.

SUPPA is a robust tool to study the local AS event level across multiple conditions. We employed it to generate seven simple AS events [alternative 5′ splice site (A5SS), alternative 3′ splice site (A3SS), skipping exon (SE), retained intron (RI), mutually exclusive exons (MXE), alternative first exon (AFE), alternative last exon (ALE)] from annotation file and quantified the AS event inclusion levels (*PSI*) in 7 preimplantation developmental stages. If the *PSI* value of AS event is in the range of 0–1 in every replicate sample of every stage, this event was identified as true AS event in this stage. The number of AS events in every developmental stage corresponding to coding gene was listed in Table 1. In mouse annotation file, a total of 58,597 AS events involved with 11,462 coding genes were identified. The ratio of coding genes occurring AS is 52.05%, which is remarkably lower than that in human annotation file (76.67%). The highest proportion of AS pattern is AFE and SE, which account for 39.3 and 23.8% of all AS events, respectively. In seven preimplantation developmental stages, the average number of AS event is 24,802 implicated with 6,877 coding genes, which is distinctly decreasing than that in annotation file (11,462). Similarly, AFE and SE are prevalent in all preimplantation developmental stages. The RI events are relatively sparse. Due to the structural complexity of MXE and limit of SUPPA, the accuracy and number of identifying MXE is poorer. Thus, we didn't take account of MXE pattern in sequence conservation analysis of AS. Isoforms generated by these AS events either encoded different protein variants or regulated the protein concentration via nonsense-mediated decay (NMD) mechanism.

**Table 1:** The number of AS events for different developmental stages.

	**A5SS**	**A3SS**	**SE**	**RI**	**MXE**	**AFE**	**ALE**	**Sum**
GTF	6,335	7,089	13,945	3,059	1,203	23,054	3,912	58,597/11,462^*^
Oocyte	2,965	3,506	7,701	1,491	394	6,910	1,129	24,096/6,741^*^
Zygote	3,098	3,684	7,876	1,545	410	7,221	1,174	25,008/6,875^*^
2-cell	3,276	3,905	8,099	1,698	409	7,387	1,211	25,985/7,147^*^
4-cell	3,089	3,636	7,426	1,662	360	6,574	1,111	23,858/6,659^*^
8-cell	3,074	3,663	7,196	1,722	326	6,003	1,004	22,988/6,624^*^
Morula	3,269	3,835	7,486	1,806	368	6,619	1,101	24,484/6,780^*^
Blastocyst	3,599	4,160	8,217	1,973	413	7,613	1,224	27,199/7,311^*^

* *The number after “/” in Sum column denotes the gene number involved with AS*.

AS can give rise to distinct protein products. If the length of alternative region of AS event is (3*n, n* = 1, 2, 3, …) bp, this AS will conserve the reading frame and only add some new amino acids. Thus, the 3D-structure and function of protein products translated from AS isoforms are similar. If the length of alternative region of AS event is (3*n* + 1) bp or (3*n* + 2) bp, this AS will shift the reading frame and change all amino acids after splice site. Thus, the function of protein products translated from AS isoforms is more variable (Roy and Penny, 2007; Kovacs et al., 2010). Here, we systematically analyzed the ability of conserved reading frame (CRF) of AS events during preimplantation developmental stages (Table 2). For the type of RI and A5SS, the average percentage of AS events with length of alternative region equals (3*n*) bp is close to that with length of alternative region equals (3*n* + 1) bp or (3*n* + 2) bp. It means that CRF ability of RI and A5SS is moderate, but the ability of alterative reading frame (ARF) is strong. If RI is widespread, the proteome will become disorder. Under evolutionary selection pressure, the number of RI pattern becomes rare in mammal transcriptome. It has been revealed that the translation of mRNA derived from ARF was often suppressed by a premature termination codon (PTC) that results in NMD of the mRNA product (McGlincy and Smith, 2008). This mechanism imposed restriction on the protein-coding ability of RI and explain that why the function of numerous RI is unclear. On the contrary, the average percentage of AS events with length of alternative region equals (3*n*) bp in type of SE and A3SS is ~48%, which is significantly higher than that with length of alternative region equals (3*n* + 1) bp or (3*n* + 2) bp (Mann–Whitney *U* test: *p* < 0.01). This finding suggested that SE and A3SS have strong CRF ability and moderate ARF ability. Thus, SE is universal across mammal genome. Besides, there isn't remarkably difference of the CRF and ARF ability between different developmental stages.

**Table 2:** The length characteristic of alternative region of AS.

	**A5SS**			**A3SS**			**SE**			**RI**		
	**%3 = 0**	**%3 = 1**	**%3 = 2**	**%3 = 0**	**%3 = 1**	**%3 = 2**	**%3 = 0**	**%3 = 1**	**%3 = 2**	**%3 = 0**	**%3 = 1**	**%3 = 2**
Oocyte	0.3838	0.3295	0.2867	0.5000	0.2496	0.2504	0.4951	0.2497	0.2552	0.3387	0.3508	0.3105
Zygote	0.3838	0.3254	0.2908	0.4891	0.2638	0.2470	0.4892	0.2541	0.2567	0.3366	0.3476	0.3159
2-cell	0.3846	0.3291	0.2863	0.4891	0.2671	0.2438	0.4843	0.2532	0.2625	0.3345	0.3321	0.3333
4-cell	0.3784	0.3305	0.2910	0.4860	0.2690	0.2451	0.4760	0.2586	0.2654	0.3273	0.3454	0.3273
8-cell	0.3709	0.3390	0.2902	0.4767	0.2722	0.2512	0.4722	0.2639	0.2639	0.3275	0.3444	0.3281
Morula	0.3741	0.3334	0.2924	0.4746	0.2751	0.2503	0.4698	0.2634	0.2668	0.3300	0.3439	0.3261
Blastocyst	0.3682	0.3331	0.2987	0.4748	0.2779	0.2474	0.4737	0.2613	0.2651	0.3300	0.3431	0.3269
DAS	0.3743	0.3073	0.3184	0.5120	0.2771	0.2108	0.4825	0.2558	0.2616	**0.3800**	0.2200	0.4000

*%3 = 0, %3 = 1 and %3 = 2 denote that the length of alternative region of AS equals (3n) bp, (3n + 1) bp, and (3n + 2) bp, respectively. The value in every cell denotes the percentage*.

Among the 5,453 genes with two or more AS events occurred at specific stage, 1,979 were in occyte, 2,068 in zygote, 2,288 in 2-cell, 2,074 in 4-cell, 1,783 in 8-cell, 2,059 in morular and 2,229 in blastocyst. The 2-cell stage has the highest number of AS genes, implicating that the transcriptome profile in 2-cell stage is more complicated. The transcript diversity before zygote was mainly originated from maternal transcripts. After zygote, the zygotic transcripts begin to synthesize. Thus, we observed an elevated AS number during zygote and 2-cell stages. Therefore, it can be proposed that AS may be activated after zygote, especially at 2-cell stage. This process is referred to as ZASA. Obviously, the time point of ZASA is in conformity with ZGA. It was known that the embryonic stem cells in blastocyst stage will be rapidly differentiated into endoderm, mesoderm and ectoderm lineages (Feng et al., 2012). A lot of regulatory proteins need to regulate this highly sophisticated differentiation process. More AS in blastocyst could provide an important source of protein diversity in this stage. Hence, the elevated AS number was observed in blastocyst (Table 1). These data suggested that the profile of AS is dynamic at different stages during preimplantation stages. Besides, we founded that the vast majority of AS events in preimplantation developmental stages is biased toward either high (>80%) or low (<20%) inclusion ratio (Figure S7), which is consistent with the previous study by Busch and Hertel (2013).

**Figure 3:**
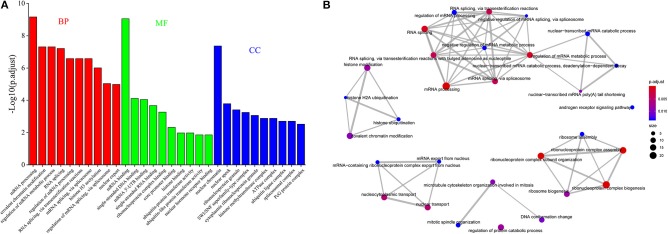
The functional analysis of genes occurring DAS. **(A)** GO enrichment analysis of a conserved gene dataset occurring AS during preimplantation development. **(B)** The network of the enriched GO-BP terms for DASGs between zygote and 2-cell stage.

Furthermore, we observed that 213 genes were expressed with multiple AS events (≥2) within every cell at all of the seven developmental stages and of which 12.68% is overlapped with splicing factor. The GO enrichment analysis showed that many genes were enriched on pre-mRNA splicing regulation (Figure 3A). We extrapolated that a conserved gene set regulating AS during preimplantation development might be found.

Moreover, by combining the transcript-level data, diiffSplice module of SUPPA was utilized to identify DAS and DASGs between different preimplantation development stages (see Materials and Methods). A total of 6,546 DASs derived from 5,610 DASGs were identified for the contrast groups of 7 preimplantation development stages. After deleting duplicates between different contrast groups, 2,269 DASs embedded in 1,060 DASGs were listed. For the seven consecutive development stages, 1,170 DAS derived from 998 DASGs were identified. After deleting duplicates between different contrast groups, 1,060 DASs embedded in 836 DASGs were listed (Table 3). It was shown that the number of DAS and DASGs from zygote to 2-cell stages is the greatest (Figure 1E). This peak point is also coincided with ZGA. Besides, we elaborated the distribution of DAS pattern in consecutive development stages (Table S7 and Figure S8). It was indicated SE and AFE are the most widespread DAS. However, the SE percentage of DAS between occyte and zygote is the highest, implying that DAS generated by SE may be important for the formation of zygote. It must be emphasized that Table 2 showed that the ability of CRF of RI events in DAS was elevated. It implied that RI may play a great role in regulatory mechanism associated with DAS events.

**Table 3:** The number of DAS and DASGs.

	**Oocyte**	**Zygote**	**2-cell**	**4-cell**	**8-cell**	**Morula**	**Blastocyst**
Oocyte	0	**281/244**	380/311	426/350	392/326	479/382	565/449
Zygote		0	**304/250**	365/291	342/283	382/318	461/376
2-cell			0	**138/120**	218/192	247/208	422/344
4-cell				0	**85/74**	126/113	310/259
8-cell					0	**115/99**	261/210
Morula						0	**247/211**
Blastocyst							0

*Cutoff: p-value ≤ 0.05 and dPSI ≥ 0.1. Bold digits is corresponding to the seven consecutive development stages*.

In addition, we found that many DASGs are significantly enriched in GO-BP terms of splicing regulation, stem cell population maintenance, ribosome assembly, histone modification, etc. Especially between zygote and 2-cell stage, a total of 83 GO-BP terms involved 122 DASGs were significantly enriched, of which the majority terms were associated with splicing regulation. For dissecting the function of enriched terms, we interwoven the top 30 most significantly enriched terms into a network with edges connecting overlapping gene sets (Figure 3B). The larger the mutually overlapping gene sets were, the more likely the terms to be clustered together. It was indicated that five functional modules were identified, of which a module was involved with histone modification, and all of the other modules were closely related to pre-mRNA splicing process. It is well-known that histone modification is a key marker of exon definition and AS regulation (Luco et al., 2011; Zhou et al., 2014). It may imply that DASGs could play important roles for AS regulation during preimplantation development.

Also, each stage-specific DEGs and DASGs were identified (Table S8). Obviously, the number of stage-specific DEGs and DASGs between 2-cell and zygotes are greatest. The KEGG pathway enrichment showed that the majority of the pathways involved in stage-specific DEGs were significantly enriched in RNA transport, spliceosome, mRNA surveillance pathway, oocyte meiosis, cell cycle, and disease, etc. The stage-specific DASGs were mainly enriched in the pathway of mRNA surveillance and hormone signaling.

### AS of Splicing Factors Associated With Pre-embryonic Development

RNA-binding proteins (RBPs) play critical roles in post-transcriptional gene regulation (PTGR), such as regulation of AS, mRNA stabilization, mRNA location, polyadenylation and translation. Gerstberger et al. (2014) manually curated 1,542 human RBPs that interact with all known classes of RNAs, described their families and evolutionary conservation across species, and analyzed their expression across tissues and their potential roles in developmental processes. The mechanism of AS involves *cis*-acting RNA elements, *trans*-acting proteins, epigenetic factors, etc. Most of these trans-acting proteins are RBPs, especially SFs (Carazo et al., 2018).

**Figure 4:**
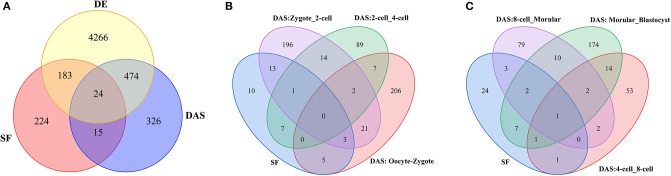
**(A)** Venn diagram of SF, DE and DASGs across seven consecutive stages of preimplantation development. SF denotes 446 splicing factors. DE denotes 4,947 DE genes in consecutive development stage. For example, DAS denotes 836 genes undergoing DAS in consecutive development stage. **(B,C)** Number of SFs at every consecutive stage of preimplantation development. SF represents 39 SFs undergoing DAS across seven consecutive stages of preimplantation development. For example, DAS: Occyte_Zygote denotes these genes undergoing DAS from occyte to zygote stages.

In this study, a total of 446 mouse splicing factors were selected for analysis based on literature mining for previously described splicing functions (Han et al., 2013; Goldstein et al., 2017), and “RNA splicing” or “splicesome”-associated Gene Ontology (GO) terms from MGI. Venn diagram displayed ~77% (342) SFs is included in human RBPs (Figure S9). We observed that about 50% (207/446) SFs are differentially expressed during preimplantation development (Figure 4A). Furthermore, 39 SFs have intersection with 836 DASGs, which means these SFs undergo self-AS in the regulatory process of mRNA processing (Figures 4B,C, Table S4). Moreover, 17 DASGs that belong to SF between zygote and 2-cell were detected. As compared to other five consecutive stage of preimplantation development, this number is the greatest, suggesting that more SFs function during ZASA especially from zygote to 2-cell stage.

**Figure 5:**
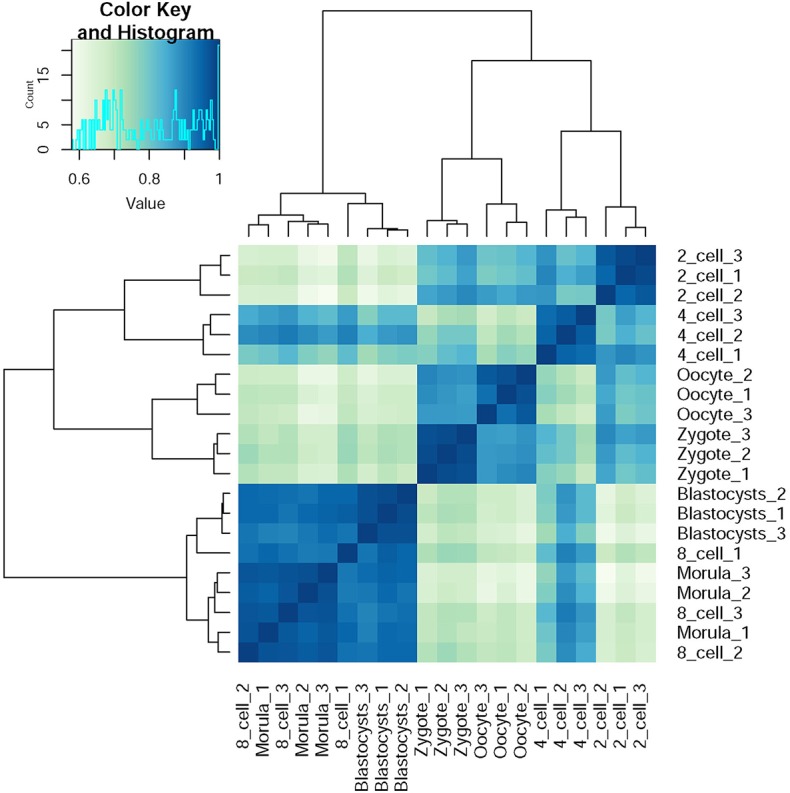
The hierarchical clustering of preimplantation development stages based on specific SFs.

SFs are pivotal factors for all AS regulation. For clarifying the specific of these SFs on preimplantation development, we performed hierarchical clustering based on Pearson Correlation coefficient of gene expression level between different developmental stages (Figure 5). Cells that clustered together were at the same developmental stages in all cases, with the exception that a morula-stage cell was interchanged with a blastocyst-stage cell. Furthermore, the developmental time series was also approximately captured from oocytes to blastocysts, as neighboring stages clustered together in the analysis as to be expected, similar to what has been previously reported by Yan et al. (2013). Only gene expression information of 39 SFs was employed, but the clustering result was good enough. This phenomenon suggested these SFs expression is specific for preimplantation development and is crucial for normal preimplantation development.

**Figure 6:**
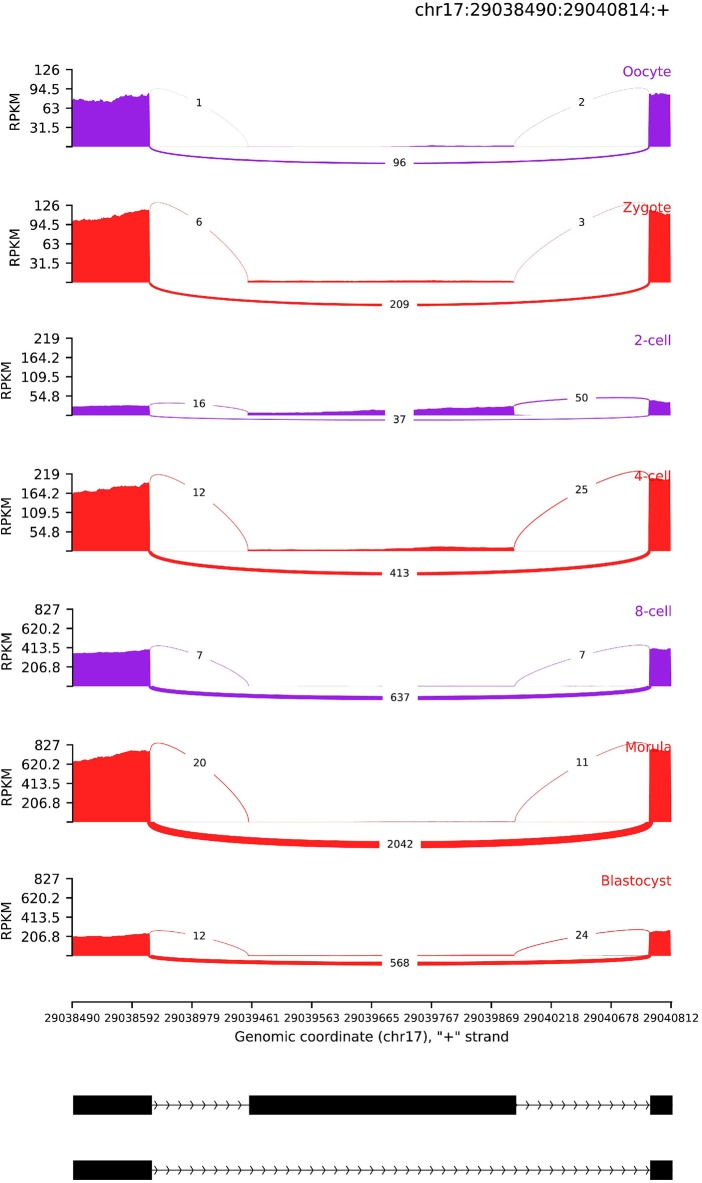
AS read coverage of SRSF3. The x-axis and y-axis denotes genomic coordinate and transcript expression level (RPKM), respectively. The black rectangle and line represent exon and intron, respectively.

SRSF3 (serine/arginine-rich splicing factor 3, alias: Srp20) is the smallest member of the SR proteins (serine-arginine-rich family of nuclear phosphoproteins) family of splicing factors. In UniProt Database, SRSF3 has two transcript isoforms (P84104–1 and P84104–2), and P84104–1 is the dominant isoform. P84104–2 is produced at very low levels due to a premature stop codon in the mRNA, leading to NMD. Interaction with YTHDC1, a RNA-binding protein that recognizes and binds N6-methyladenosine (m6A)-containing RNAs, promotes recruitment of SRSF3 to its mRNA-binding elements adjacent to m6A sites, leading to exon-inclusion during AS. It was revealed that SRSF3 is essential for mouse development. If SRSF3 was knocked out, blastocyst formation was prevented and caused death of preimplantation embryos at the morular stage (Jumaa et al., 1999). SRSF3 is also essential for later developmental decisions, such as those in B-cell development. In consistent with this conclusion, we observed that gene expression level of SRSF3 is remarkable elevated in morular and blastocyst stages compared to in oocytes and early stages of embryonic development. Besides, SRSF3 has the lowest expression level in 2-cell stage. It was revealed the majority maternal RNAs of SRSF3 are degraded in 2-cell stage (Figure 6). The gene expression level of SRSF3 escalates after this stage. It was indicated that majority transcripts of SRSF3 are synthesized by zygotic activation.

Furthermore, we also identified an exon skipping AS event in SRSF3, of which isoforms are translated P84104–1 and P84104–2. The detailed AS profile of SRSF3 can be viewed in Figure 6. The P84104–1 skips an exon (chr17:29039454–29039909) and the transcript expression level is higher, particularly in morular and blastocysts. Because the degradation of maternal RNAs, exon inclusion level *PSI* in 2-cell stage is significantly lower than other developmental stages. After 2-cell stage, transcript expression level of P84104–1 is compensated by ZGA. The isoform P84104–1 is dominant in morular and blastocyst stage. The blastocyst is the first developmental stage with known differentiated cell lineages, suggesting that P84104–1 isoform of SRSF3 is essential for initiating this early genetic programme. This result also implied that the AS of SRSF3 is popular in pre-embryonic development stages (Sen et al., 2013). The relative concentration of RNA-binding activator and repressor of splicing machinery is an important regulator of splice-site recognition (Wang et al., 2015). SR proteins and hnRNPs (heterogeneous nuclear ribonucleoproteins) are RNA-binding activator and repressors, respectively. As a SR protein, the concentration of SRSF3 can be modulated by self-splicing. Then, splice site recognition of other genes which have the potential binding site of SRSF3 can be regulated by SRSF3.

### The Identification of ISs in Consecutive Stages of Preimplantation Development

Over 1,000 DAS events embedded into 836 genes were identified during seven consecutive development stages. Every DAS gene included more than one isoform. We used Time-Series Isoform Switch (TSIS) program to detect ISs, where the expression level of different isoforms is reversed during preimplantation development (Guo et al., 2017). As input file of TSIS, the abundance (TPM) of 3,096 transcripts involved with all DAS was extracted from transcript expression matrix. A total of 212 significant (*p* < 0.05) ISs that embraced two transcript isoforms were identified in 836 unique DASGs. TSIS determines the two time points between which a significant isoform switch occurs, and consistent with the DE and DAS, the majority (62.26%) occurred between 2 cell/zygote and 4 cell/2-cell (Figure 1E and Table S9). Thus, in response to ZGA, there are crest of ISs between 2 cell/zygote.

**Figure 7:**
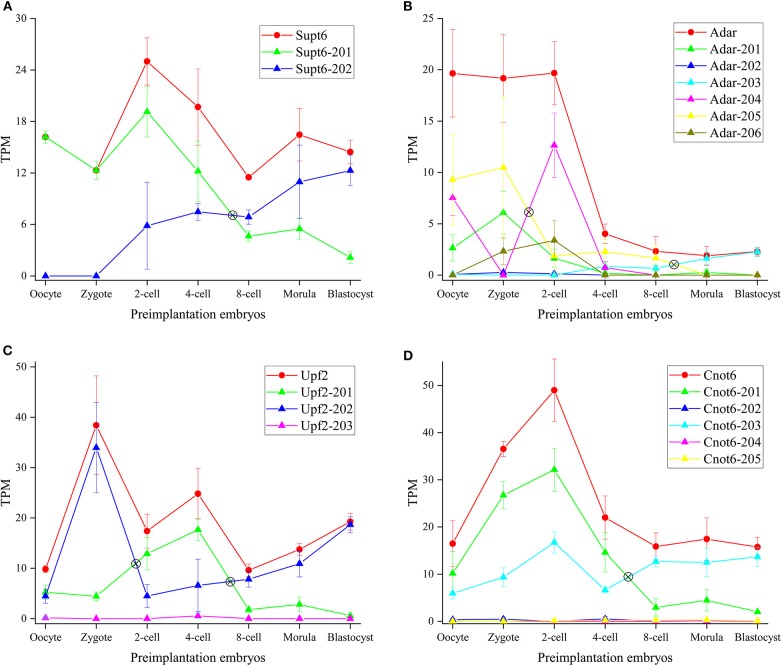
Expression profiles of DASGs. **(A–D)** Represent the gene and transcript expression profiles of *Supt6, Adar, Upf2* and *Cnot6*, respectively. The y-axis denotes TPM (Transcripts Per Kilobase Million). The symbol ⊗ denotes switch point. The red line denotes gene expression level and other color lines denote transcript expression level.

*Supt6* (alias: *Spt6* or *Supt6h*) is a transcription elongation factor which binds histone H3 and plays a critical role in the regulation of transcription elongation and mRNA processing. It produces two different transcript isoforms, of which *Supt6*-201 can be translated into protein with 1,726 residues, and protein product of *Supt6*-202 has not been detected yet (Hubbard et al., 2002). The transcript abundance can be modulated by SE of exon 6 in *Supt6*-202. *Supt6* showed a significant IS between 4-cell and 8-cell (Figure 7A). It suggested protein factor translated from *Supt6*-201 plays a crucial role in ZGA, and *Supt6*-202 may play a role in post-implantation development. During ZGA, *Supt6* can promote activation of transcriptional elongation via *Tat*, and enhance the transcription elongation by RNA polymerase II (RNAPII). *Supt6* can also recruit mRNA export factors (*Alyref*/*Thoc4, Exosc10*) and histone-lysine N-methyltransferase (*Setd2*) to assist mRNA splicing, mRNA export and elongation/splicing-coupled H3K36 methylation by forming *Supt6*:IWS1:CTD complex. Xu et al. (2019) revealed *Setd2* plays a vital role in establishing the maternal epigenome and exerts important impacts for preimplantation. The expression profile of *Setd2* is similar with *Supt6* (Figure S10), which demonstrated *Setd2* and *Supt6* may locate in the same regulated network. *Setd2* generates 10 transcripts, which were respectively annotated as protein-coding, nonsense mediated decay or no-protein isoforms. We observed transcript abundance of *Setd2*-201, *Setd2*-204, and *Setd2*-210 is dominant and the trend of these transcripts is identical with *Setd2*. The *Setd2*-201 and *Setd2*-210 was already labeled as protein-coding isoforms. However, the protein product from *Setd*2-204 has not been found so far. It implied that *Setd2*-204 may exert important function in previously unknown pathways during preimplantation development.

*Adar* enzyme can catalyze the hydrolytic deamination of adenosine to inosine (A-to-I) in double-stranded RNA (dsRNA). It may participate in biological regulation in a number of ways that include mRNA translation, pre-mRNA splicing, RNA stability, genetic stability and RNA structure-dependent activities, and so on (The UniProt Consortium, 2019). *Adar* modulates trans-acting factors involved in the AS machinery by affecting splicing regulatory elements (SREs) within exon (Solomon et al., 2013). Here, three transcript isoforms with TPM = 0 in 21 cells were dropped, and expression profiles of 6 transcript isoforms were analyzed (Figure 7B). Qiu et al. (2016) constructed A-to-I RNA editome during early human embryogenesis and demonstrated *Adar* expression and A-to-I RNA editing level remained relatively stable until 4-cell stage, but dramatically decreased at 8-cell stage, continually decreased at morula stage. Similar to human embryogenesis, in mouse embryogenesis, we demonstrated *Adar* expression level was stable and remarkably elevated until 2-cell stage, but sharply decreased at 4-cell stage, continually decreased until blastocysts stage. It was deduced that A-to-I RNA editing level is also parallel with *Adar* expression level in mouse embryogenesis. García-López et al. (2013) has revealed that A-to-I editing in microRNAs in mouse preimplantation embryos is mediated by *Adar*. We speculated A-to-I RNA editing is dynamically changed during preimplantation development in a stage-specific fashion and plays a vital role in activating zygotic genes. Furthermore, a clearly IS was identified between zygote and 2-cell. Expression level of *Adar*-204 sharply increased and that of *Adar*-201 and *Adar*-205 significantly decreased from zygote to 2-cell stage. It indicated that (1) on the condition that *Adar* expression level is relatively constant, abundance of transcript isoforms is variable during preimplantation development; (2) transcript isoforms executing dominant regulating role is different during different preimplantation development stages. Besides, the lincRNA (*Adar*-206) was expressed during ZGA. As non-coding RNA, *Adar*-206 may execute special regulatory role during ZGA.

AS is a common form of post-transcriptional regulation in metazoan. Concomitantly, it has been estimated that over one third of the AS events also create aberrant transcript isoforms that trigger NMD pathway (Bao et al., 2016). As a RNA surveillance mechanism, NMD machinery eliminates aberrant transcript harboring PTC (premature termination codon) signal and plays an essential role in safeguarding the transcriptomic fidelity in the cell. The NMD machinery includes three core factors: *Upf1, Upf2*, and *Upf3*, in addition to *Smg1-7*, which are highly conserved in eukaryotes (Schweingruber et al., 2013). In recent years, some studies demonstrated that *Upf2*-dependent NMD pathway performs an essential role in Spermatogenesis, tissue development, disease (Thoren et al., 2010; Nguyen et al., 2014; Bao et al., 2016). To explore whether the NMD pathway plays a role in mouse embryogenesis, we plotted expression profiles of *Upf2* (Figure 7C). It was showed that *Upf2* expression level is fluctuant and reached peak at zygote stage. We can extrapolate NMD pathway is critical for preimplantation development, especially for fertilization. By analyzing IS, we observed that *Upf2-*202 isoform is dominant during fertilization and blastocyst formation, and *Upf2*-201 isoform is more prevalent during ZGA. Besides, as lincRNA, the expression level of *Upf2*-203 is very lower.

*Cnot6* is a subunit of the CCR4-NOT core transcriptional regulation complex, which is one of the major cellular mRNA deadenylases. It is linked to various cellular processes including transcription and translation regulation, mRNA degradation, miRNA-mediated repression, cell proliferation, cell survival and cellular senescence. This gene has 5 transcripts, of which *Cnot6*-201and *Cnot6*-203 are translated into proteins and *Cnot6*-202, *Cnot6*-204, and *Cnot6*-205 are labeled as lincRNA. Previous work revealed that *Cnot1* and *Cnot3* are critical for deadenylation of maternal mRNA during mouse early embryogenesis (Ma et al., 2015; Liu et al., 2016). The expression profile showed *Cnot6*-201 and *Cnot6*-203 is overwhelming expressed product during preimplantation development (Figure 7D). Before 8-cell stage, *Cnot6*-201 is main transcript product. On the contrary, *Cnot6*-203 isoform is dominant after 8-cell stage. It suggested *Cnot6*-201 executes main role during ZGA, and *Cnot6*-203 plays important role in development of inner cell mass and blastocyst formation.

Moreover, we also plotted expression profiles of *Msh4* and *Luc7l*. *Msh4* is involved in meiotic recombination and segregation of homologous chromosomes at meiosis (Figure S11). A differential SE event and IS were identified between zygote and 2-cell. Obviously, these transcript isoforms is maternal-derived and its expression decreases with preimplantation development. The *Luc7l* encodes a putative RNA-binding protein similar to the yeast *Luc7p* subunit of the U1 snRNP splicing complex that is normally required for 5′ splice site selection. The expression of *Luc7l* and its 9 transcript isoforms exhibit oscillated patterns with preimplantation development, suggesting that *Luc7l* is likely to play a role during ZGA (Figure S12).

## Discussion

During preimplantation development from an occyte, cells progressively develop toward to the blastocyst as zygotic genome is activated. It has been extensively studied that gene expression level is spatial-temporally dynamic during early embryonic development (Fan et al., 2015; Schultz et al., 2018). Recently, some evidence indicated that AS could correlate closely with preimplantation development, suggesting a key role for splicing in regulating early embryonic development (Revil et al., 2010; Yan et al., 2013). However, previous results about the diversity and function of AS in early embryonic development were mainly based on a few isolated examples. In this study, we carried out genome-wide comprehensive analysis on seven preimplantation developmental stages to capture the dynamic changes of gene expression and AS during early stages of embryonic development.

The accurate identification and quantification of transcripts and genes paves the way of downstream omics analysis. Here, the performance of different quantifying strategies was compared (Table S10). As alignment-free transcript quantification, the Salmon outperforms HISAT2 belonged to alignment-based transcript quantification. Since our goal was to measure the abundance of the known coding-gene isoforms, we selected Salmon to perform transcript and gene quantification in 21 scRNA-seq datasets. Identifying the set of DEGs across different developmental stages is an important goal in this study. DESeq2 for analyzing count-based NGS data can accurately detect DEGs in bulk RNA-seq data. We observed that the number of DEGs identified by DESeq2 was greater than that by Seurat (Table S10). However, the comparison of different quantifying strategy between 2-cell and zygote showed 95.83% DEGs identified by Salmon + Seurat had been included in those identified by Salmon + DEseq2 (Figure S13). We postulated that if DESeq2 were applied directly to scRNA-seq data, the false-positive rate would be relatively high. In contrast, the result derived from Seurat would be more accurate (Freytag et al., 2018). Thus, we employed Seurat to detect DEGs during preimplantation development.

In the time-course analysis of preimplantation embryo, 4,952 DEGs and 836 DASGs were respectively detected in consecutive seven developmental stages. Over 10% DEGs were differentially alternatively spliced. It suggested that the crosstalk between transcription and AS regulation might occur during preimplantation development. In concert with major ZGA in mouse preimplantation embryo (Abe et al., 2018), DEGs between 2-cell and zygote achieved the maximum, especially up-regulated genes. Functional enrichment analysis of DEGs revealed that with the initiation of transcription and translation, splicing machinery may also be assembled during ZGA. It was well-known that co-transcriptional splicing is ubiquitous for long mammalian genes (Luco et al., 2011). The fact that transcription and splicing machinery is simultaneously established during ZGA may indicate co-transcriptional splicing maybe universal in preimplantation development. Based on differentially expressed genes during zygote to 2-cell stages, Zeng and Schultz (2005) employed Ingenuity Pathway Analysis (IPA) to identify 25 regulatory networks implicated with ZGA. The most remarkable network is composed of 35 genes and centered on *Myc*. By filtering the DEGs between zygote and 2-cell stages, we identified 34 up-regulated genes embedded this network. Out of the 34 genes, 21 genes are above the threshold (adjust *p* value ≤ 0.05 and |log_*e*_*FC*| ≥ 0.25). Most genes in this network belong to ribosomal genes. This is consistent with protein synthesis and ribosome biogenesis being two major biological themes that emerge from zygote to 2-cell embryos. Besides, we constructed the top DEGs dataset between 2-cell and zygote stages including 134 top up-regulated and 152 top down-regulated genes, and deciphered the biological function. This dataset was dependable and provided a guideline for decoding the ZGA mechanism from experiment. We also detected that the number of DEGs between 4-cell and zygote stages was larger than that between 2-cell and zygote stages. It confirmed zygotic genes were activated until 4-cell stage during mouse preimplantation development. By characterizing the frequency distribution of maternal and zygotic genes, the conclusion that ZGA includes minor ZGA and major ZGA was verified.

On average, 24,802 AS events, which involved in 6,877 multi-exon protein-coding genes, were identified in every preimplantation developmental stage. The gene number occurring AS is remarkably lower than that in annotation file. This result can be mainly caused by the lower transcript complexity of preimplantation embryos, the limitation of sequencing depth, the imperfect annotation file and the defective of tools. We investigated the CRF and ARF of AS patterns and found the CRF ability of SE was significantly stronger than that of RI. It is well-known that AFE can change the gene expression level by modulating promoter activity. Thus, the percentage of the AFE and SE are dominant and this tendency is conserved in seven developmental stages. By counting the gene number occurring AS events, we found the AS profile is dynamic at different preimplantation developmental stages and the gene number with AS in 2-cell is higher than other stages. It was concluded that the time point of ZASA was coincided with ZGA and AS was activated around ZGA. Besides, a conserved gene set composed of 213 genes was constructed, which is expressed in every cell of all stages with multiple AS events and regulates AS during preimplantation development.

By identifying and analyzing DAS and DASG, we found the number of DAS and DASGs from zygote to 2-cell stages was the greatest. This result once again demonstrated that ZASA may be coupled with ZGA. During ZGA, a mass of regulated proteins are recruited to regulate gene activation. DAS of pre-mRNA can provide more diversely regulated proteins, which ensure that ZGA is executed successfully (Hamatani et al., 2004; Revil et al., 2010; Park et al., 2018). The functional enrichment analysis demonstrated that many DASGs may play important roles in splicing regulation. For DASGs between zygote and 2-cell stage, 5 functional modules closely related to pre-mRNA splicing process were hunted. It can be inferred that DASGs may be key regulator of AS during preimplantation development. This result also verified that AS may be activated with ZGA from the perspective of potential biological function and pathway.

As trans-acting proteins, SFs execute critical roles in AS. Over 50% SFs are differentially expressed and 39 SFs are differentially spliced during mouse preimplantation development. Especially from zygote to 2-cell, 17 SFs were differentially spliced. This finding showed SFs preform more function during ZASA especially from zygote to 2-cell stage. Furthermore, only using 39 SFs spliced differentially, almost all of samples can be clustered correctly. It demonstrated expression profiles of SFs are specific for different preimplantation development stages. To take SRSF3 as an example, we elaborated the dynamic changes of gene and transcript isoforms coverage during preimplantation development. Gene expression differences and AS of SFs affect the splicing modulation of a large number of targeted AS events, suggesting the existence of a regulatory cascade that SFs may regulate AS by DE and AS of their own gene transcripts during preimplantation development.

Expression level of transcript isoforms always is hidden by gene expression. Although the gene expression is relatively constant, the dominant transcript isoform is variable during time-series in early embryonic development. We identified 212 ISs in 836 DASGs where the expression level of different isoforms is reversed during preimplantation development. It must be emphasized that the crest of ISs number occurs between 2 cell/zygote. This result indicated the role of ISs during ZGA and once again confirmed that the ZASA and ZGA are synchronous. We characterized the expression profiles of gene and their transcript isoforms during 7 developmental stages and predicted the regulatory function of every transcript isoforms. *Supt*6 performs regulation function in transcription elongation and mRNA processing. We unveiled *Supt6*-201 and *Supt6*-202 may play pivotal roles in ZGA and post-implantation development, respectively. Furthermore, we investigated the crosstalk between *Supt*6 and *Setd*2, and showed *Setd2*-204 may exert important function in previously unknown pathways during preimplantation development. This provided a new insight to decoding the *Setd*2. By charactering expression profile of *Adar* and their transcripts, we proposed that A-to-I RNA editing level is dynamic during preimplantation development and play a vital role in activating zygotic genes. Moreover, it was uncovered that the lincRNA (*Adar*-206) exerts special regulatory role during ZGA. After analyzing IS feature of *Upf2* implicated with NMD pathway, the dominant isoform is identified at every developmental stage. This will facilitate researchers to clarify the NMD mechanism. As a major cellular mRNA deadenylases, the *Cnot6* expression level is significantly increased during ZGA. We can infer from the remarkable IS that *Cnot6*-201 performs key role during ZGA, and *Cnot6*-203 may play vital role in development of inner cell mass and blastocyst formation. In summary, unraveling regulatory role of DASGs during embryogenesis from transcript abundance profiles provided a new way for decoding the mystery of preimplantation development.

Overall, the dynamic atlas of DE, AS, and DAS over preimplantation development was established and was comprehensively analyzed. It was inferred that splicing factors could auto-regulate AS by self-DE and self-AS during preimplantation development. Over 200 ISs which may play crucial roles during early embryogenesis were identified. Importantly, we uncovered that ZASA is coincided with ZGA and verified that AS is coupled with transcription during preimplantation development in mouse. This study provided valuable resource and specific functional predictions for further targeted experimental validations to elucidating the regulated mechanisms of embryogenesis and early embryotic development.

## Data Availability Statement

All datasets generated for this study are included in the article/Supplementary Material.

## Author Contributions

YX analyzed the data, wrote, reviewed, and edited the manuscript. WY, HM, and ZL constructed the dataset. GL contributed to discussing and reviewing the manuscript. XC constructed the regulatory network. HZ, XZ, and JL discussed the results. LC and MZ supervised the study.

### Conflict of Interest

The authors declare that the research was conducted in the absence of any commercial or financial relationships that could be construed as a potential conflict of interest.

## Supplementary Material

Table S1 The basic information of dataset.

Table S2 Transcript expression matrix of coding genes.

Table S3 Count Matrix of coding genes.

Table S4 Splicing factors.

Table S5 GO enrichment of DE genes between 2-cell and Zygote.

Table S6 GO enrichment of DE genes between 4-cell and Zygote.

Table S7 The distribution of DAS patterns in consecutive development stages.

Table S8 The number of stage-specific DEGs and DASGs.

Table S9 Isoform switches (ISs) identified in 836 unique DASGs.

Table S10 Comparison for different detecting methods of DE genes.

Figure S1 The heatmap showing the expression levels (*TPM* value) of DEGs across seven consecutive stages of preimplantation development. The row names are the name of DEGs.

Figure S2 The heatmap showing the inclusion levels (*PSI* value) of DAS events across seven consecutive stages of preimplantation development. The row names are the name of DAS events.

Figure S3 GO-MF and GO-CC enrichment analysis of up-regulated and down-regulated genes between 2-cell and zygote stages. The dotplots only showed the top 15 most significant terms.

Figure S4 The gene expression atlas of significantly down-regulated genes in different preimplantation development stages. The gene expression level was normalized by Seurat (see Materials and Methods).

Figure S5 Functional enrichment analysis of the top DE genes between 2-cell and zygote stages.

Figure S6 GO-BP, GO-MF and GO-CC enrichment analysis of up-regulated and down-regulated genes between 4-cell and zygote stages. The dotplots only showed the top 15 most significant terms.

Figure S7 The inclusion ratio distribution of AS events in zygote.

Figure S8 The distribution of DAS pattern between consecutive development stages.

Figure S9 Venn diagram between SFs of mouse and human RBPs.

Figure S10 Expression profile of *Sted2*. The *y*-axis denotes TPM (Transcripts Per Kilobase Million). The symbol ⊗ denotes switch point. The red line denotes gene expression level and other color lines denote transcript expression level.

Figure S11 Expression profile of *Msh4*. The *y*-axis denotes TPM (Transcripts Per Kilobase Million). The symbol ⊗ denotes switch point. The red line denotes gene expression level and other color lines denote transcript expression level.

Figure S12 Expression profile of *Luc7l*. The *y*-axis denotes TPM (Transcripts Per Kilobase Million). The symbol ⊗ denotes switch point. The red line denotes gene expression level and other color lines denote transcript expression level.

Figure S13 Comparison of different quantifying strategy between 2-cell and zygote. Transcript quantification methods include alignment-free transcript quantification-Salmon and alignment-based transcript quantification-Hisat2. Identifying methods of DE genes include DEseq2 and Seurat.
