# Supplementary material for: Dynamic Alternative Splicing During Mouse Preimplantation Embryo Development
Source: Front Bioeng Biotechnol. 2020 Feb 7;8:35. doi: 10.3389/fbioe.2020.00035 (PMC7019016; doi:10.3389/fbioe.2020.00035)
Supplement: Table S7 — The distribution of DAS patterns in consecutive development stages. [file Table_7.DOCX]

**The distribution of DAS patterns in consecutive development stages**

|  | A5SS | A3SS | SE | RI | MXE | AFE | ALE | Sum |
| --- | --- | --- | --- | --- | --- | --- | --- | --- |
| Oocyte-Zygote | 39 | 47 | 110 | 12 | 1 | 58 | 14 | 281 |
| Zygote_2-cell | 58 | 43 | 85 | 9 | 4 | 94 | 11 | 304 |
| 2-cell_4-cell | 25 | 17 | 39 | 10 | 4 | 39 | 4 | 138 |
| 4-cell_8-cell | 17 | 15 | 21 | 3 | 0 | 27 | 2 | 85 |
| 8-cell_Morula | 26 | 23 | 32 | 7 | 2 | 22 | 3 | 115 |
| Morula_Blastocyst | 32 | 44 | 90 | 15 | 2 | 59 | 5 | 247 |
