# Supplementary material for: Dynamic Alternative Splicing During Mouse Preimplantation Embryo Development
Source: Front Bioeng Biotechnol. 2020 Feb 7;8:35. doi: 10.3389/fbioe.2020.00035 (PMC7019016; doi:10.3389/fbioe.2020.00035)
Supplement: Table S8 — The number of stage-specific DEGs and DASGs. [file Table_8.DOCX]

**The number of stage-specific DEGs and DASGs**

|  | DEGs | DASGs |
| --- | --- | --- |
| Zygote/Oocyte | 631 | 207 |
| 2-cell/Zygote | 1014 | 183 |
| 4-cell/2-cell | 324 | 78 |
| 8-cell/4-cell | 272 | 42 |
| Morula/8-cell | 23 | 61 |
| Blastocyst/Morula | 828 | 151 |
