# Supplementary material for: Dynamic Alternative Splicing During Mouse Preimplantation Embryo Development
Source: Front Bioeng Biotechnol. 2020 Feb 7;8:35. doi: 10.3389/fbioe.2020.00035 (PMC7019016; doi:10.3389/fbioe.2020.00035)
Supplement: Table S10 — Comparison for different detecting methods of DE genes. [file Table_10.DOCX]

**Comparison for different detecting methods of DE genes**

|  | **Salmon+DESeq2** | | | **Hisat2+DESeq2** | | | **Salmon+Seurat** | | | **Hisat2+Seurat** | | |
| --- | --- | --- | --- | --- | --- | --- | --- | --- | --- | --- | --- | --- |
|  | Down | Up | DE | Down | Up | DE | Down | Up | DE | Down | Up | DE |
| Zygote/Oocyte | 1305 | 1226 | 2531 | 1153 | 1144 | 2297 | *814* | *593* | *1407* | 702 | 560 | 1262 |
| 2-cell/Zygote | 2248 | 3154 | 5402 | 1916 | 2937 | 4853 | *920* | *1311* | *2231* | 785 | 1239 | 2024 |
| 4-cell/2-cell | 2104 | 1604 | 3708 | 1819 | 1398 | 3217 | *531* | *423* | *954* | 493 | 332 | 825 |
| 8-cell/4-cell | 2029 | 1949 | 3978 | 1740 | 1733 | 3473 | *427* | *511* | *938* | 399 | 447 | 846 |
| Morula/8-cell | 437 | 206 | 643 | 438 | 249 | 687 | *39* | *27* | *66* | 35 | 25 | 60 |
| Blastocyst/Morula | 1676 | 1601 | 3277 | 1368 | 1319 | 2687 | *930* | *839* | *1769* | 833 | 777 | 1610 |

* DESeq2: *p_val_adj* <=0.05 & $\left| {log}_{2}( FC) \right|\geq1$ Seurat: *p_val_adj* <=0.05 & $\left| avg\_lnFC \right|\geq0.25$
