# Supplementary figures and images for: Dynamic Alternative Splicing During Mouse Preimplantation Embryo Development
Source: Front Bioeng Biotechnol. 2020 Feb 7;8:35. doi: 10.3389/fbioe.2020.00035 (PMC7019016; doi:10.3389/fbioe.2020.00035)

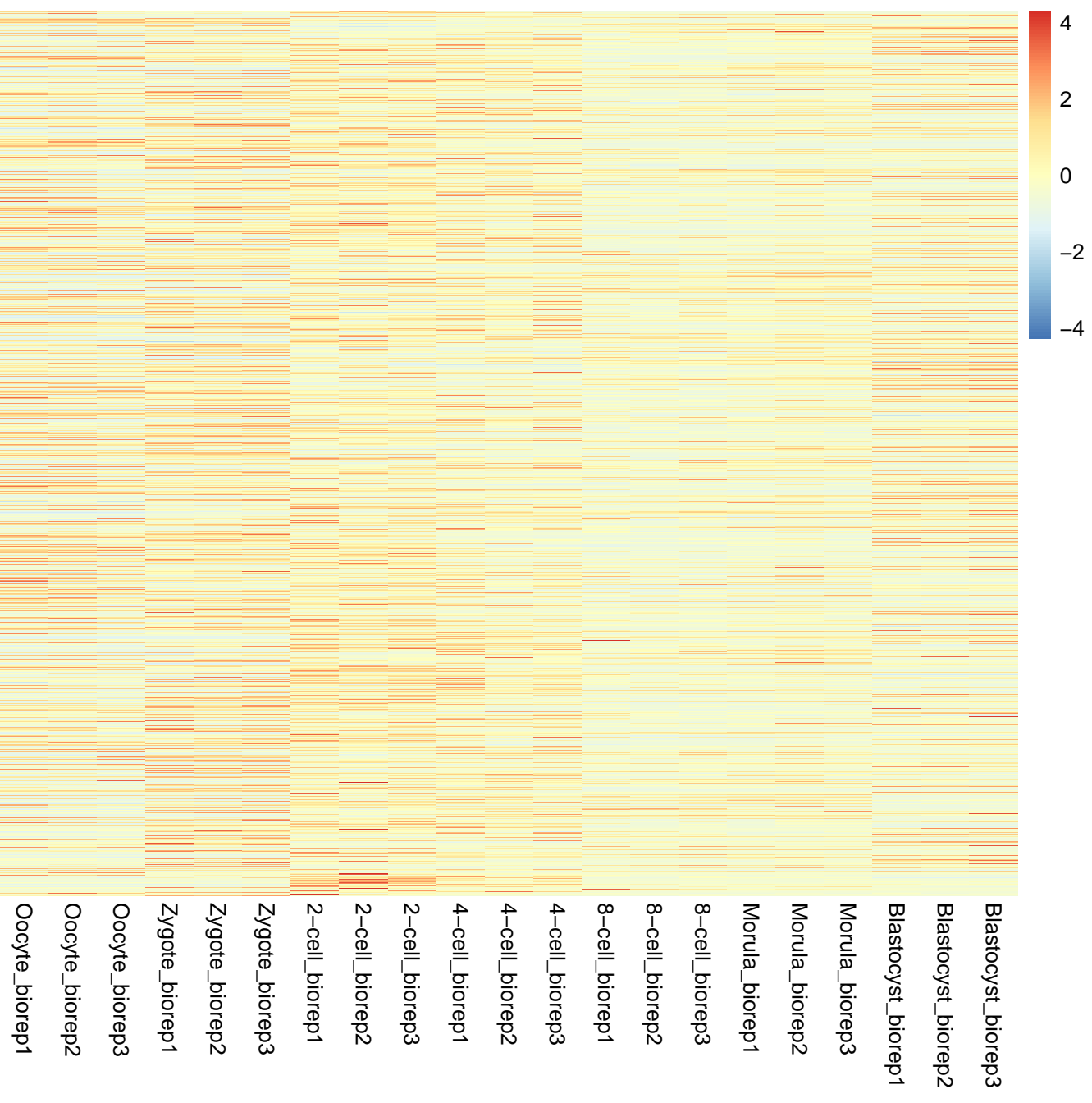

Supplement: Figure S1 — The heatmap showing the expression levels (TPM value) of DEGs across seven consecutive stages of preimplantation development. The row names are the name of DEGs. [file Data_Sheet_1.PDF]

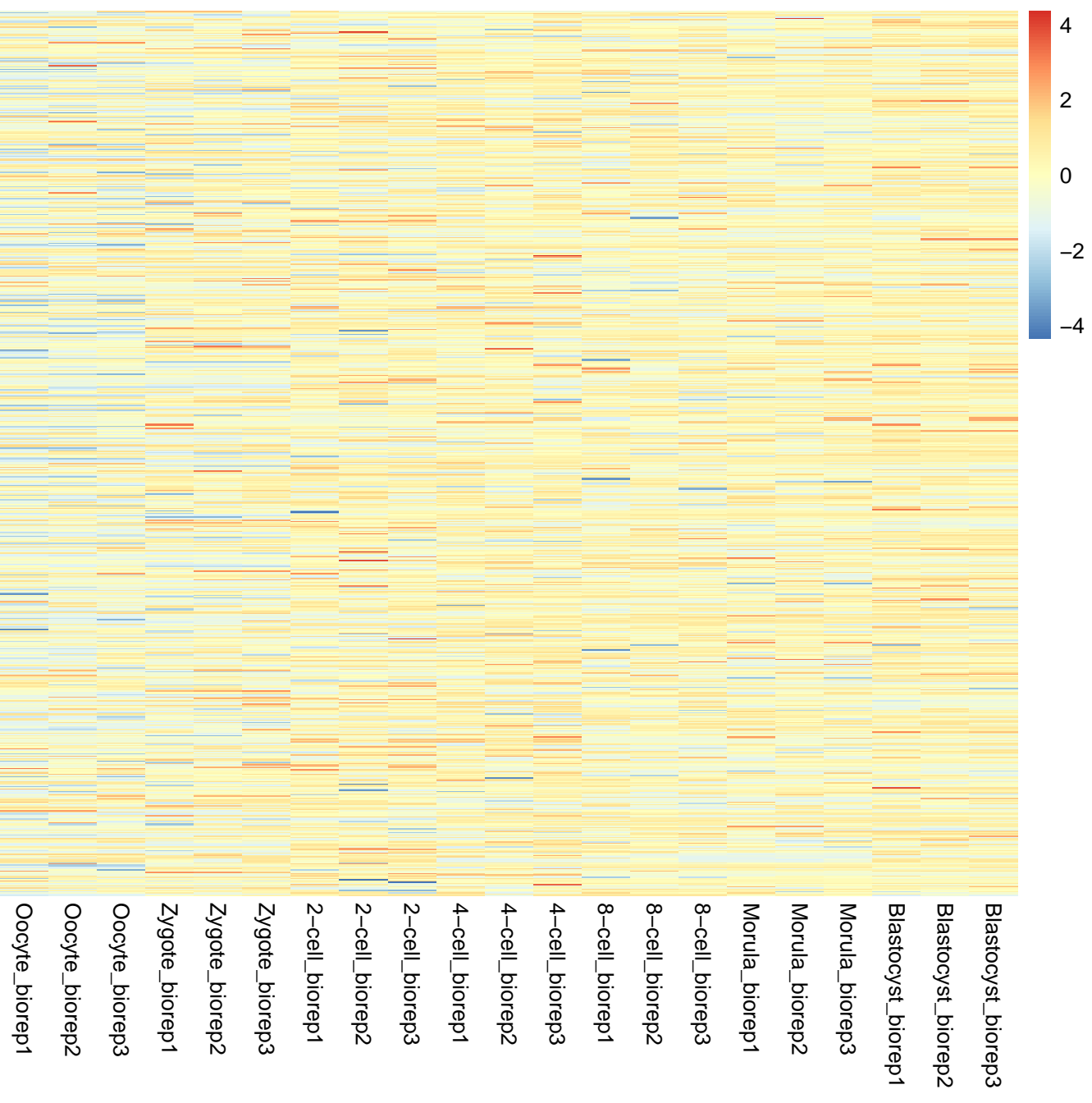

Supplement: Figure S2 — The heatmap showing the inclusion levels (PSI value) of DAS events across seven consecutive stages of preimplantation development. The row names are the name of DAS events. [file Data_Sheet_2.PDF]

2-cell\_Zygote\_Up\_BP

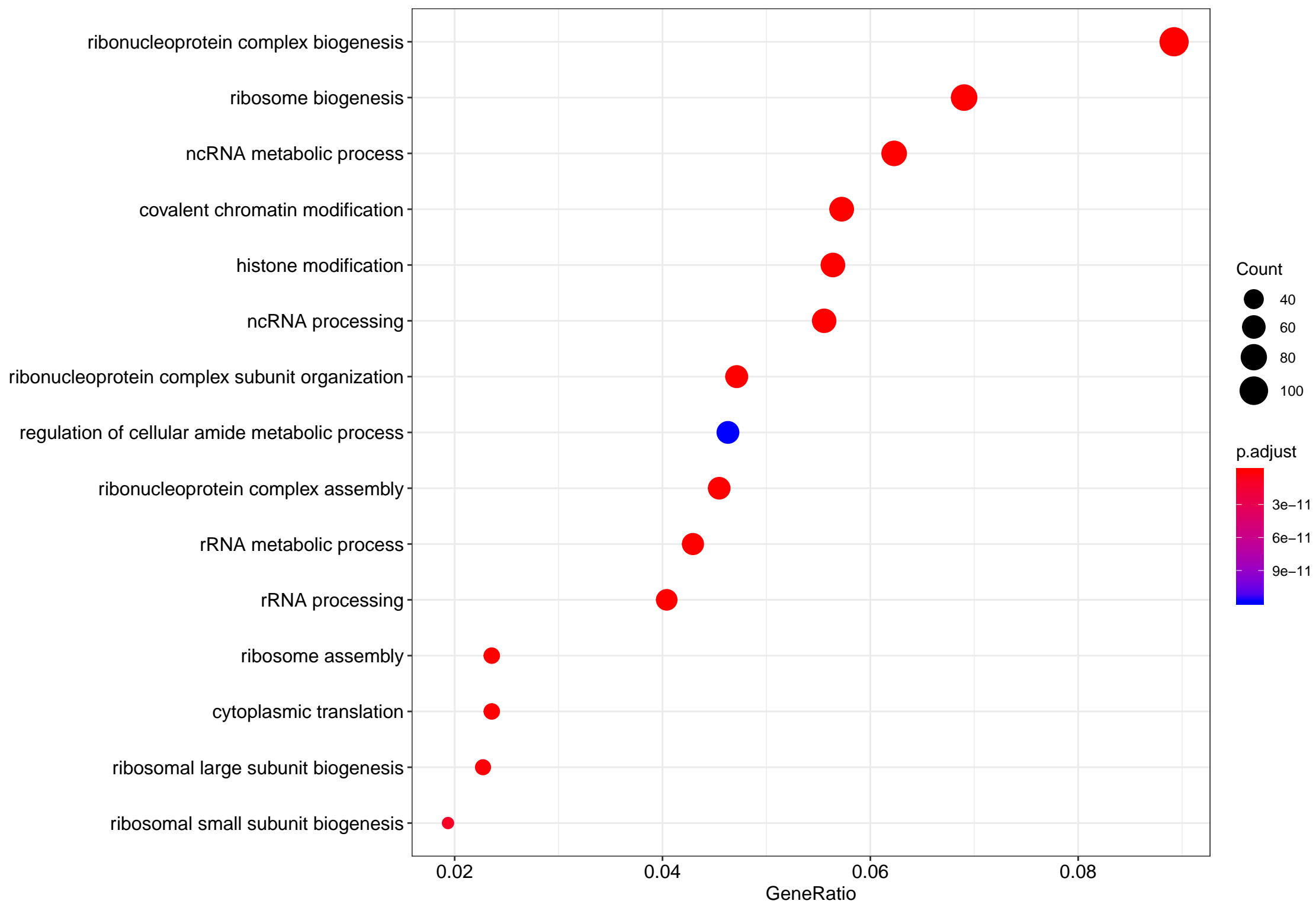

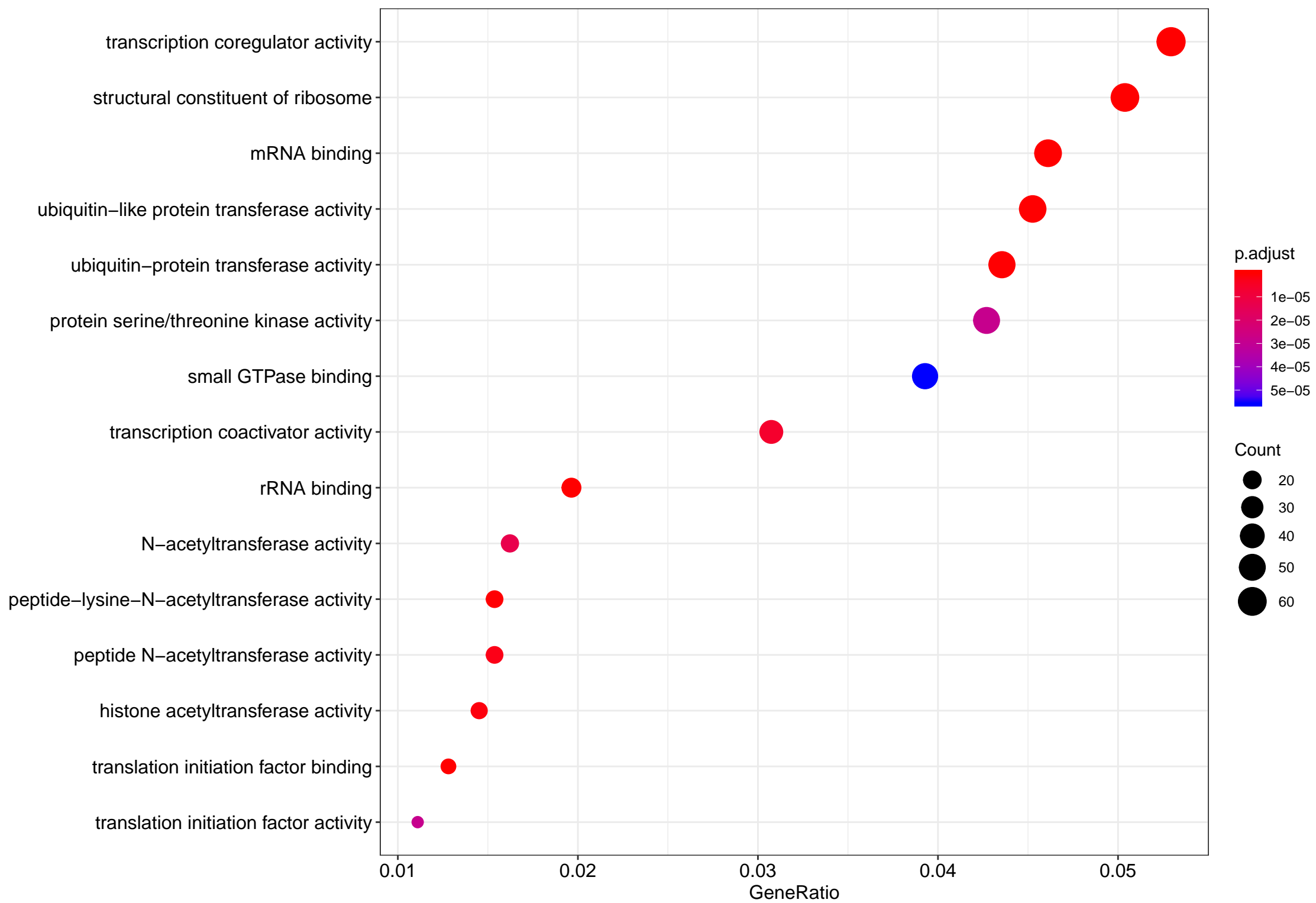

2-cell\_Zygote\_Up\_CC

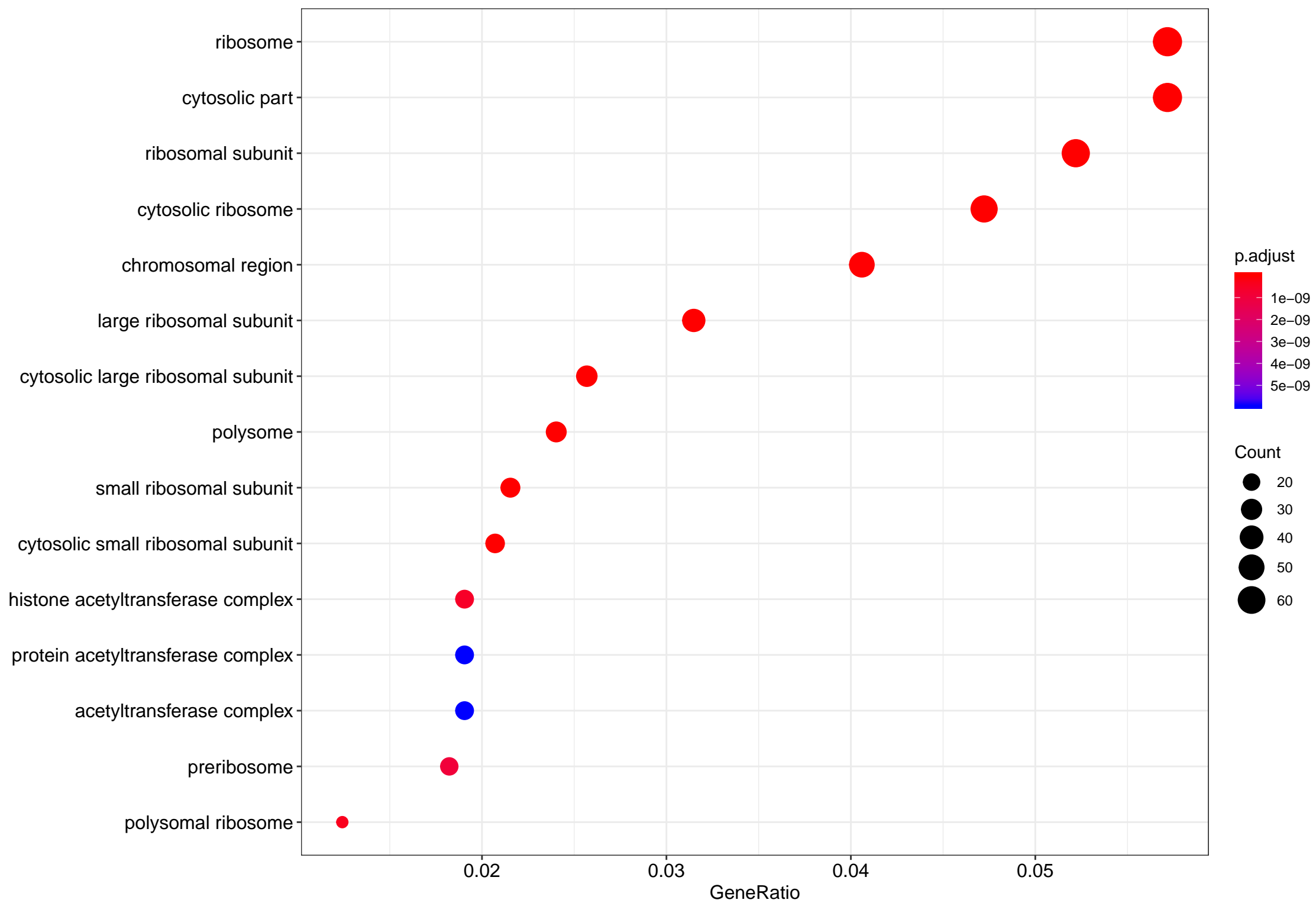

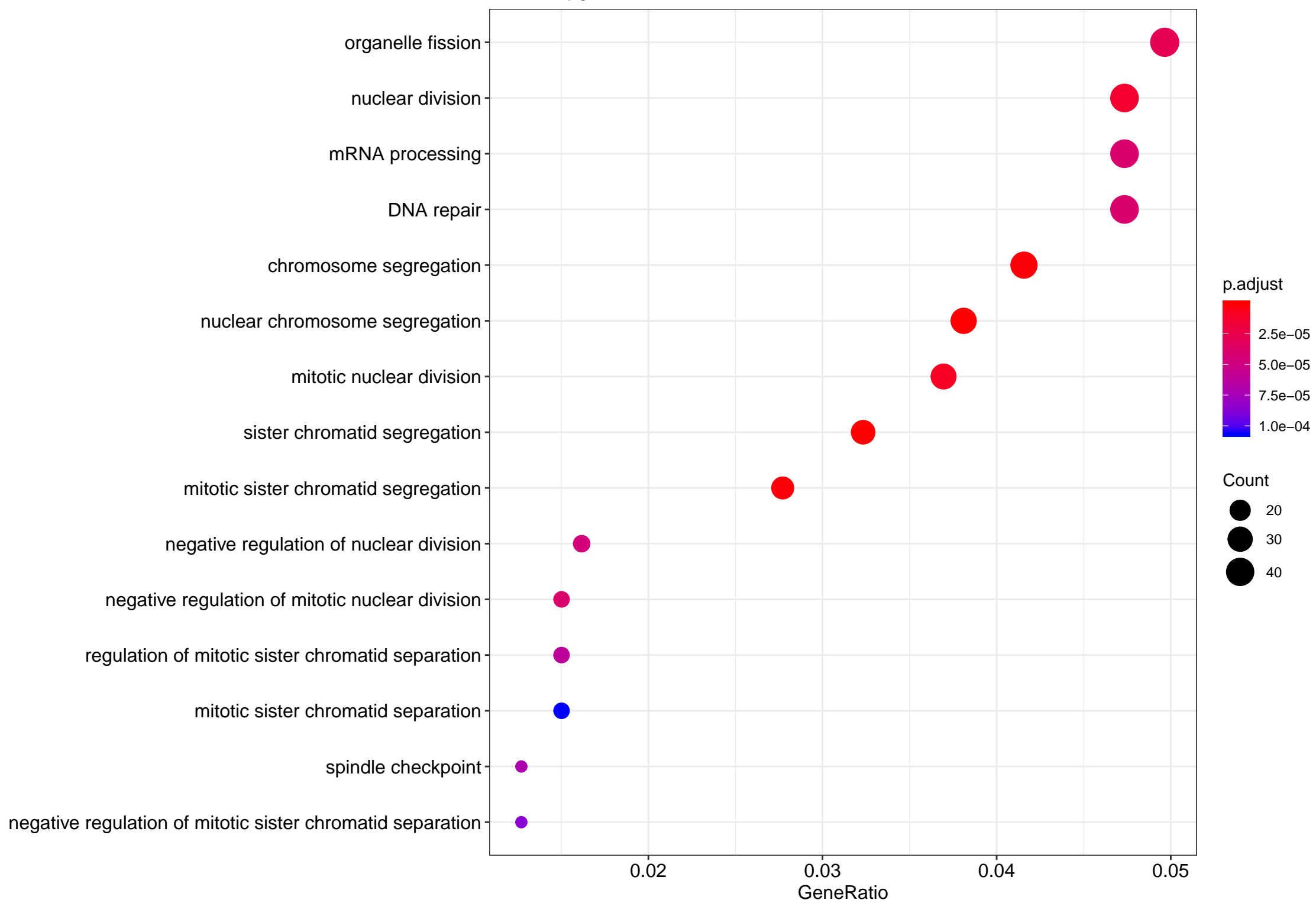

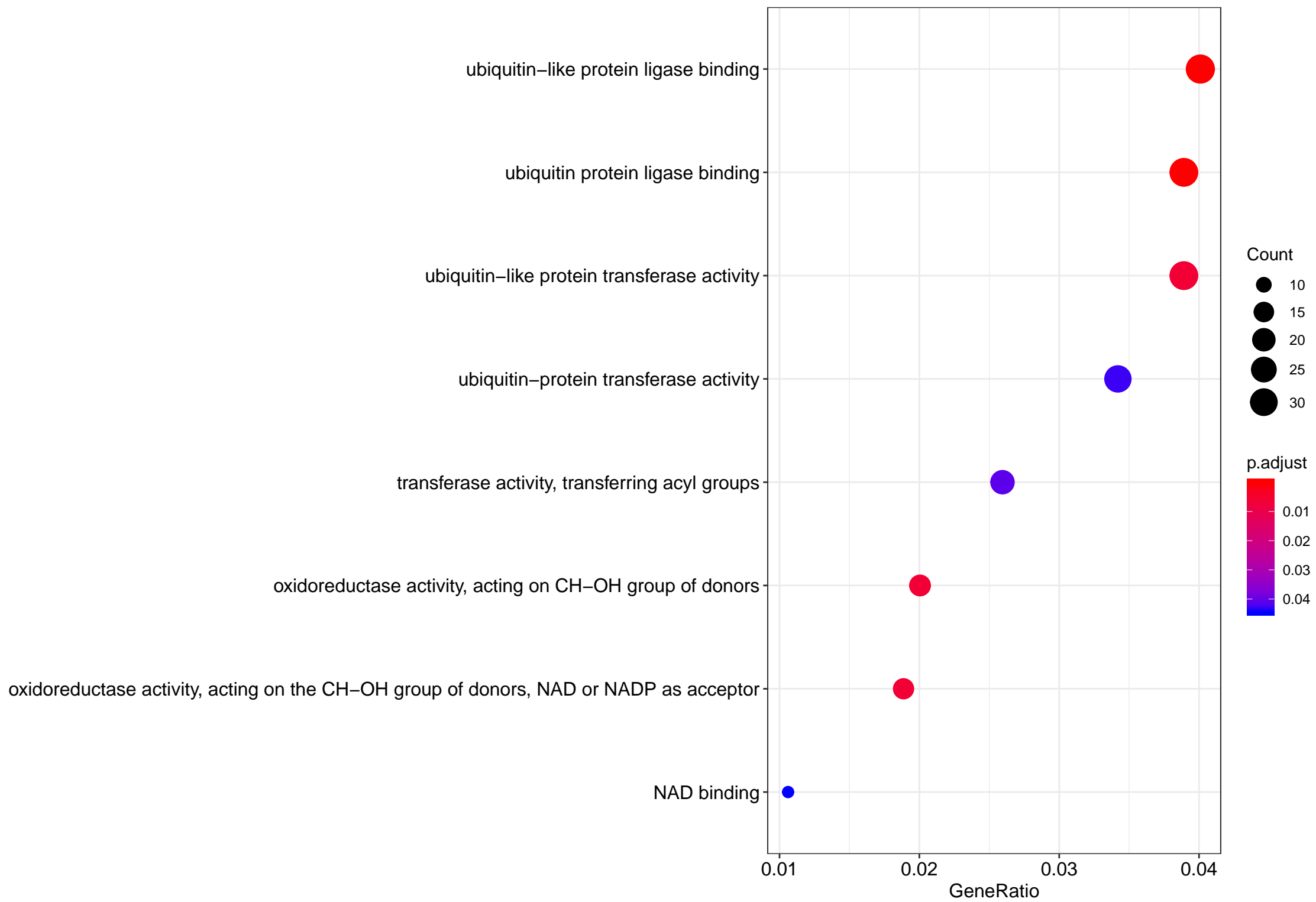

# 2-cell\_Zygote\_Down\_CC

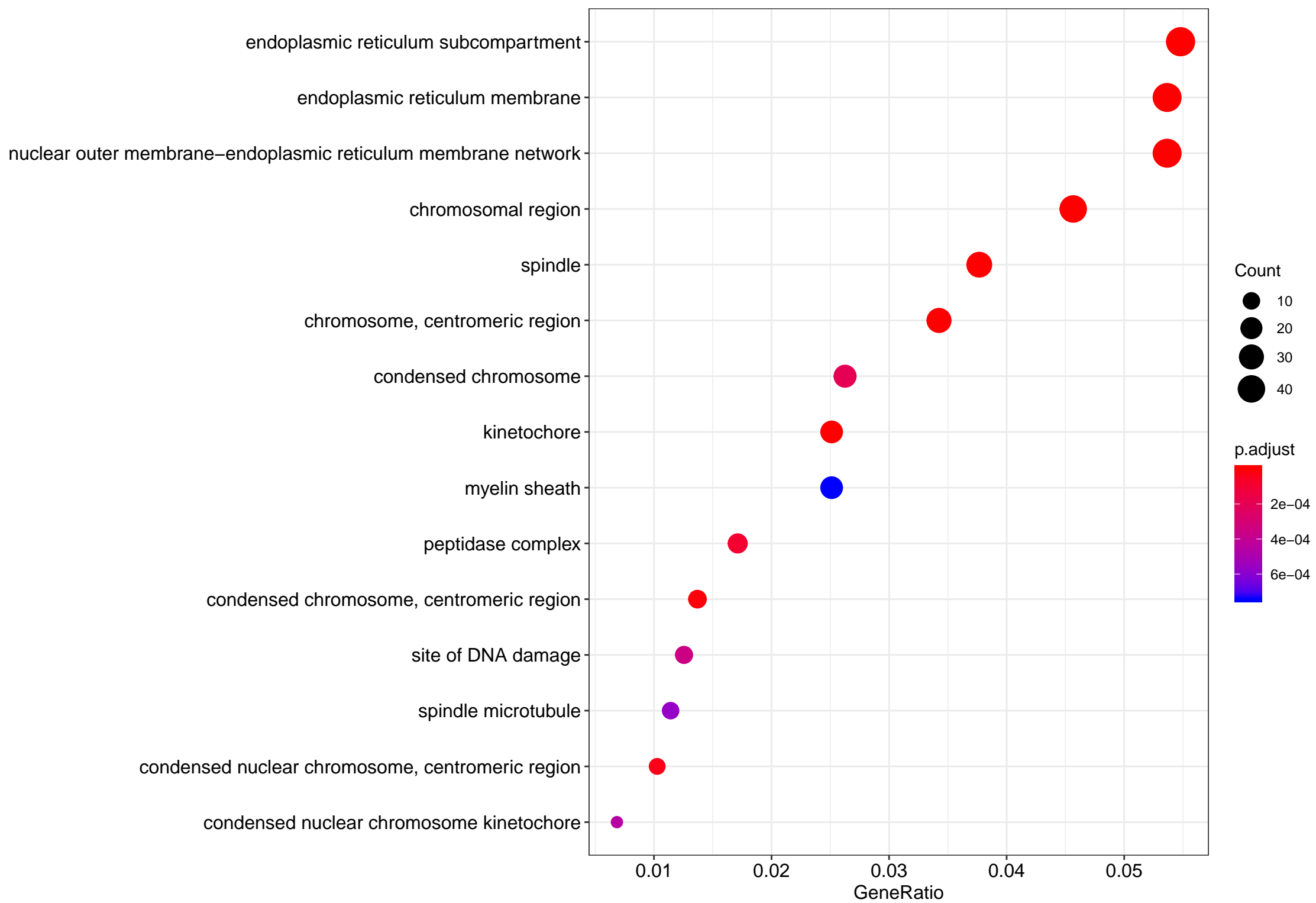

Supplement: Figure S3 — GO-MF and GO-CC enrichment analysis of up-regulated and down-regulated genes between 2-cell and zygote stages. The dotplots only showed the top 15 most significant terms. [file Data_Sheet_3.PDF]

## 4-cell\_Zygote\_Up\_BP

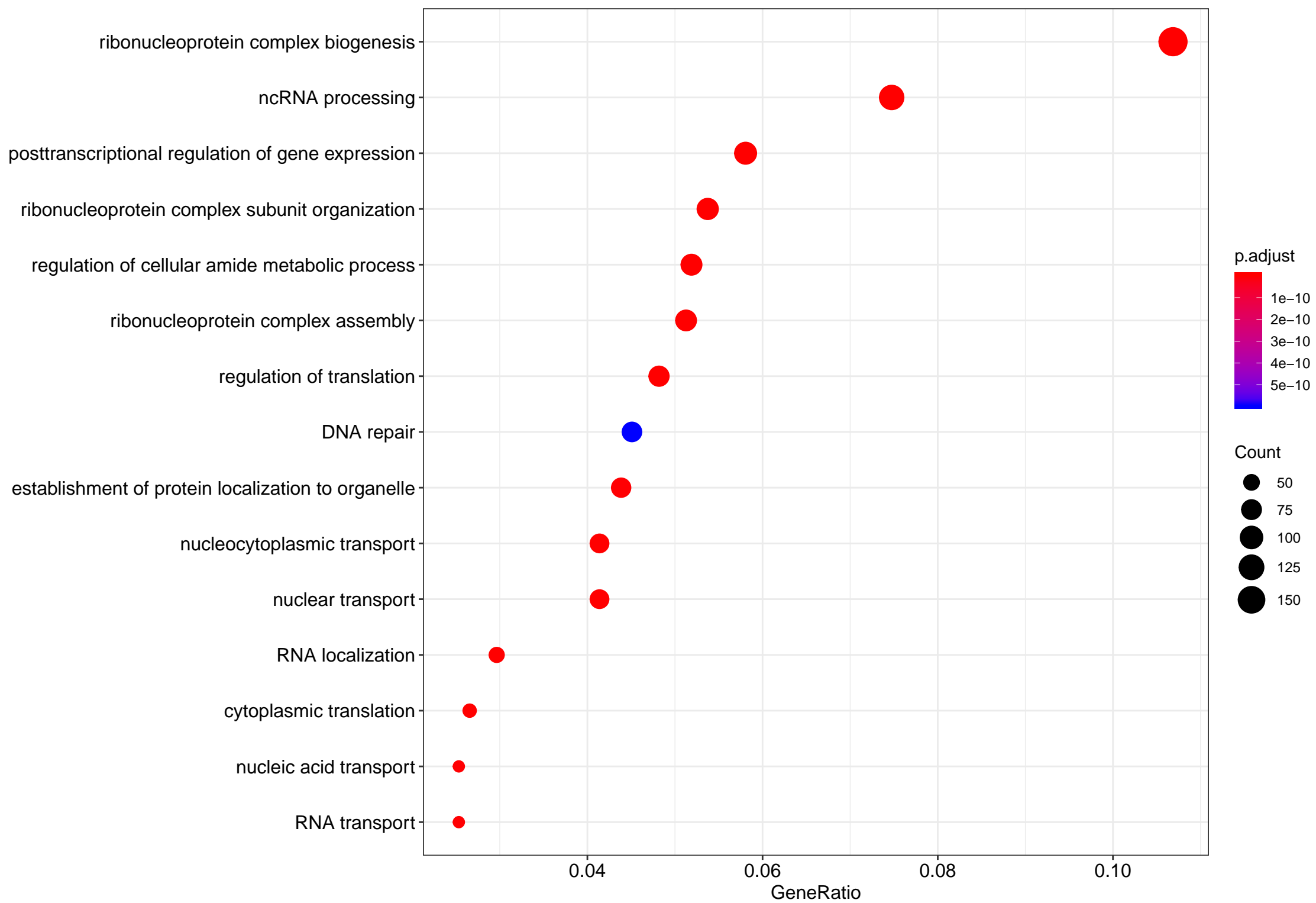

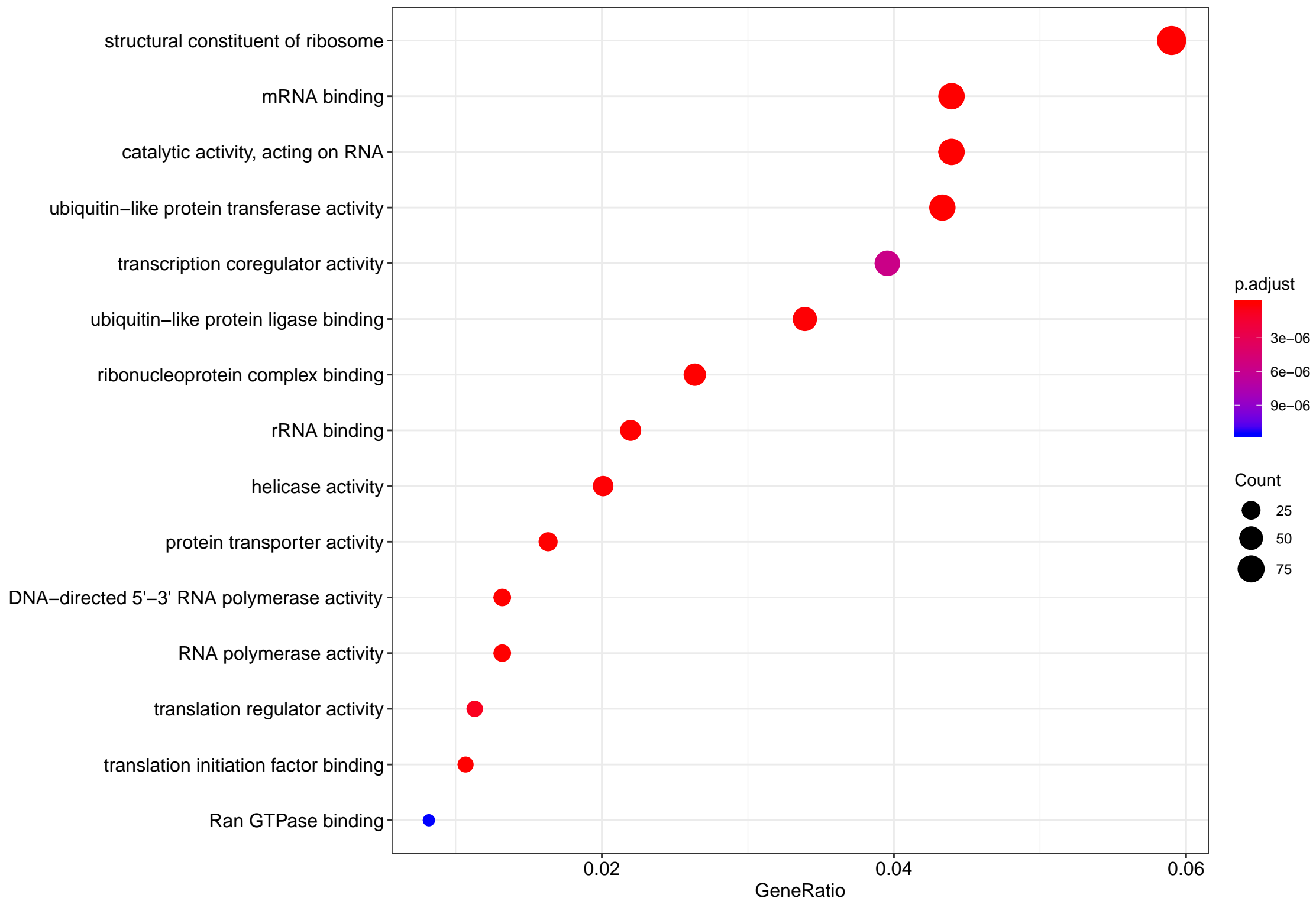

# 4-cell\_Zygote\_Up\_CC

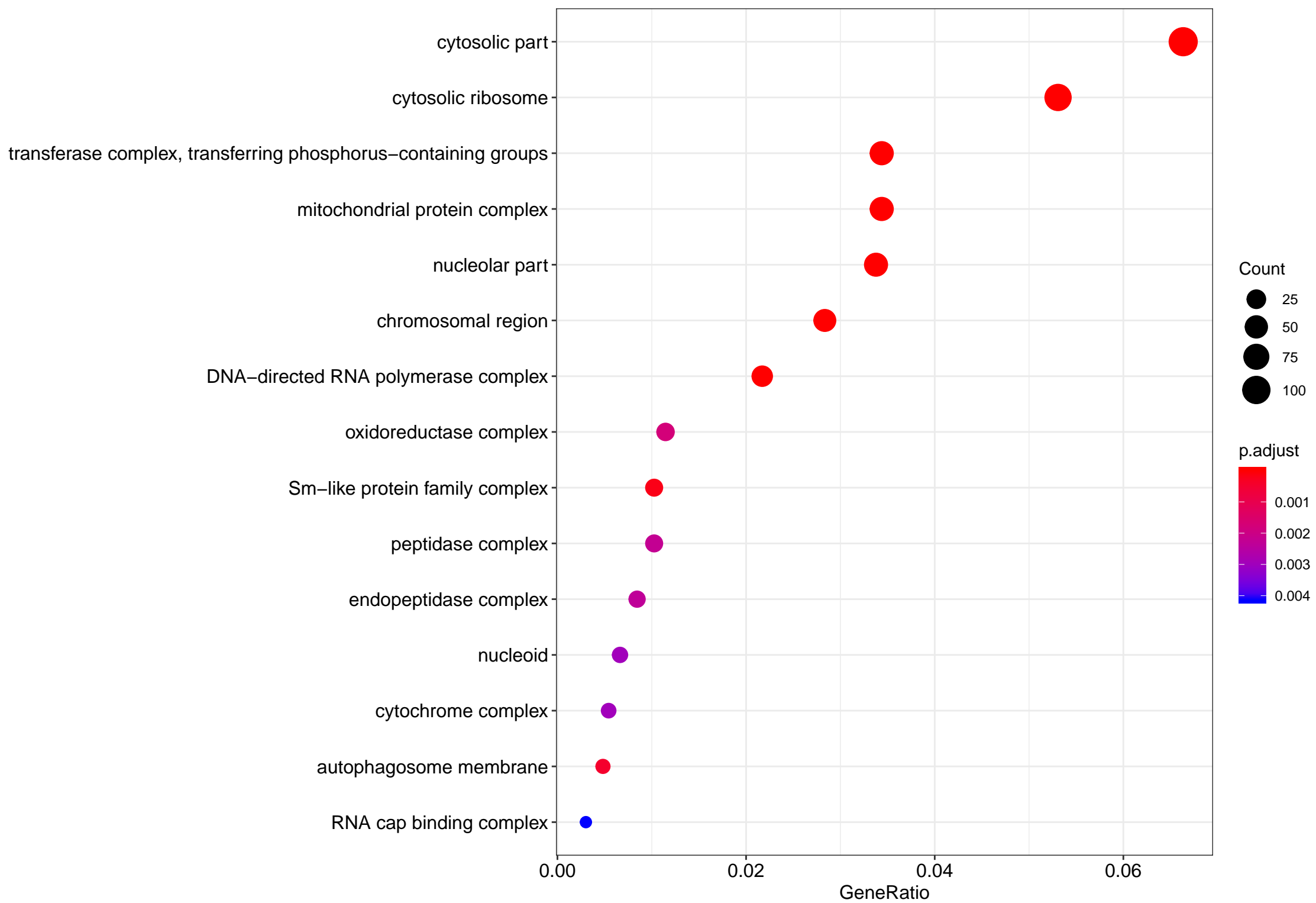

4-cell\_Zygote\_Down\_BP

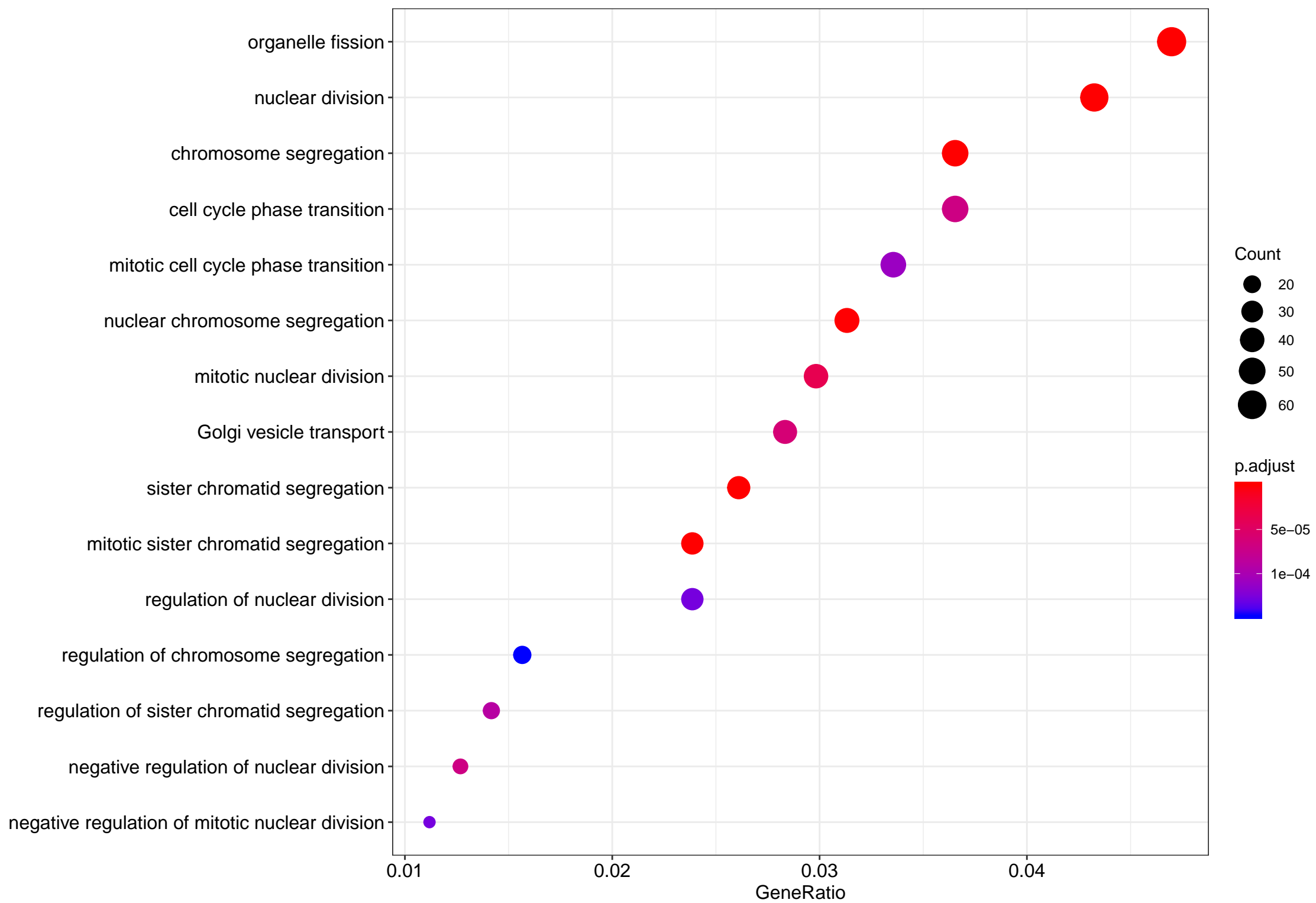

## 4-cell\_Zygote\_Down\_MF

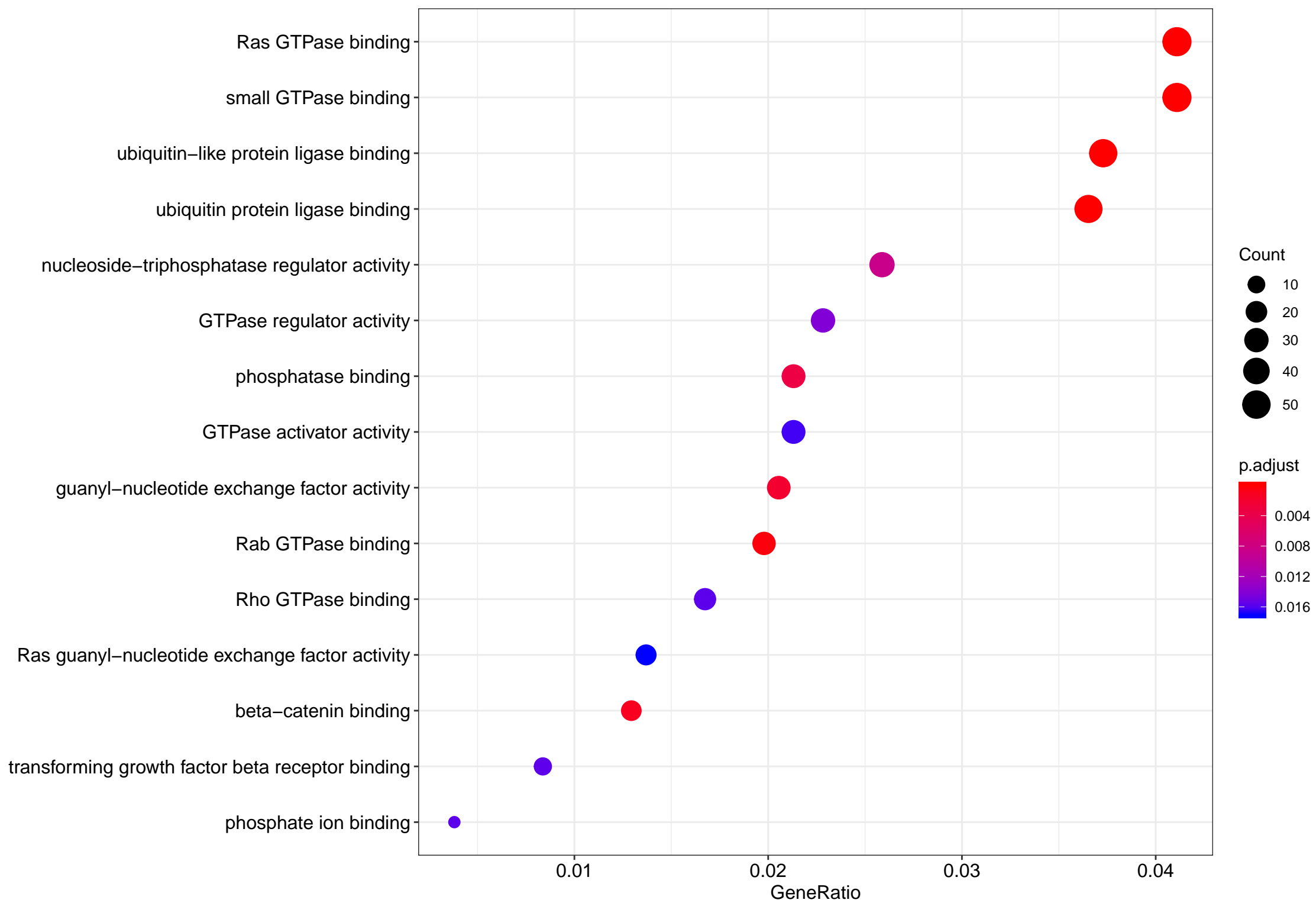

# 4-cell\_Zygote\_Down\_CC

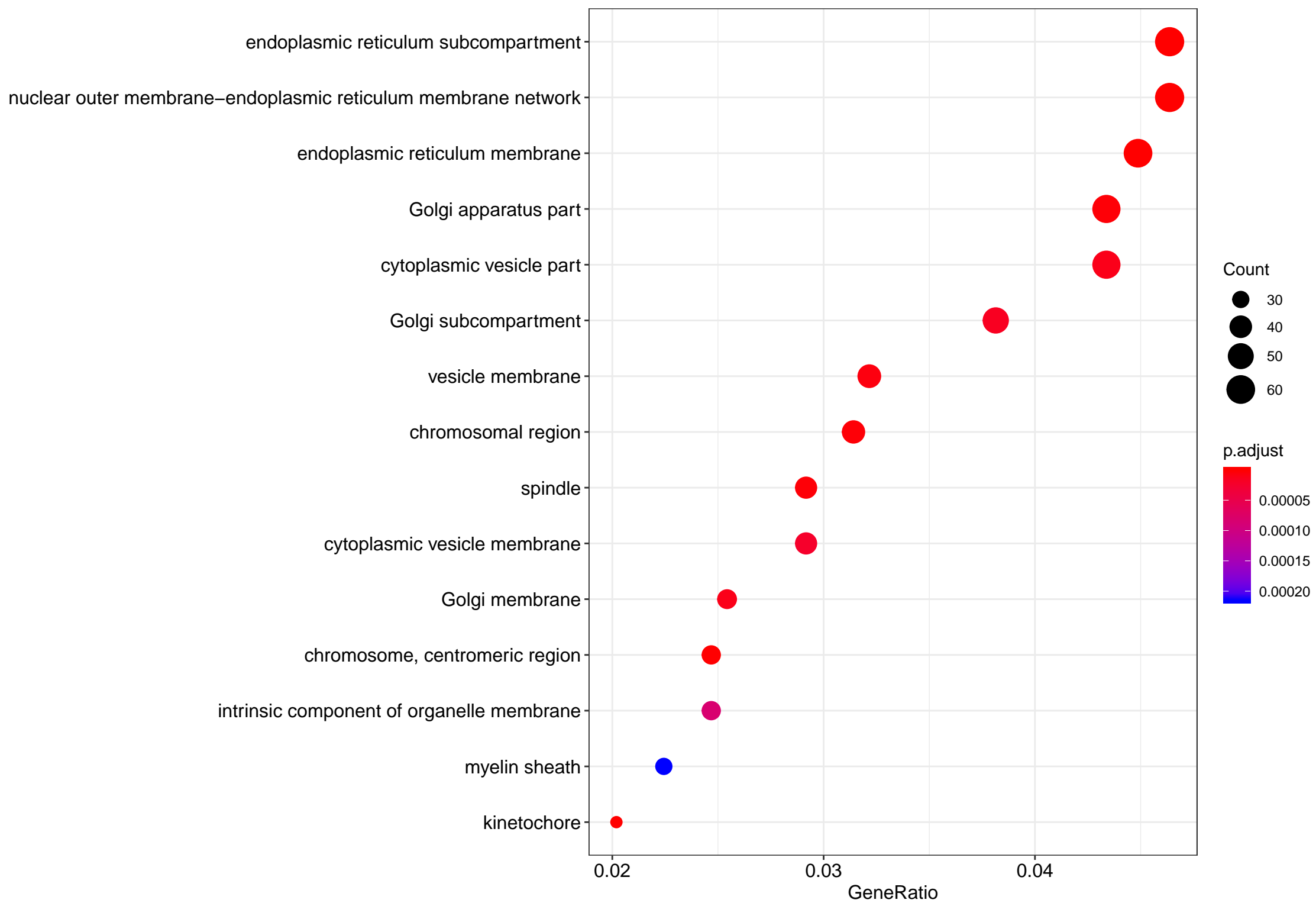

Supplement: Figure S6 — GO-BP, GO-MF and GO-CC enrichment analysis of up-regulated and down-regulated genes between 4-cell and zygote stages. The dotplots only showed the top 15 most significant terms. [file Data_Sheet_6.PDF]

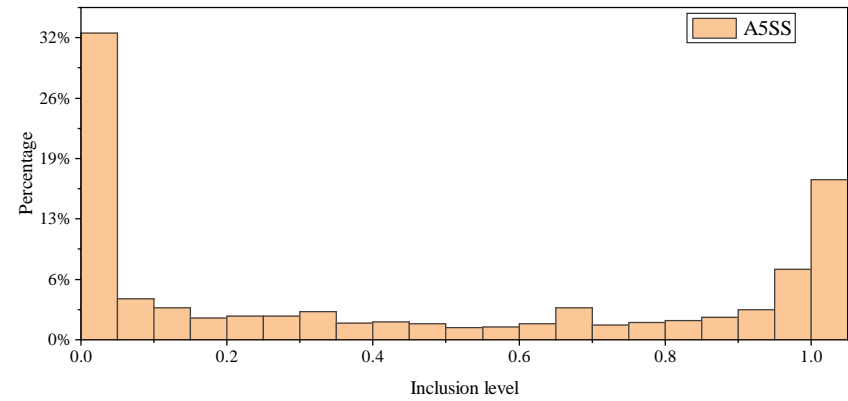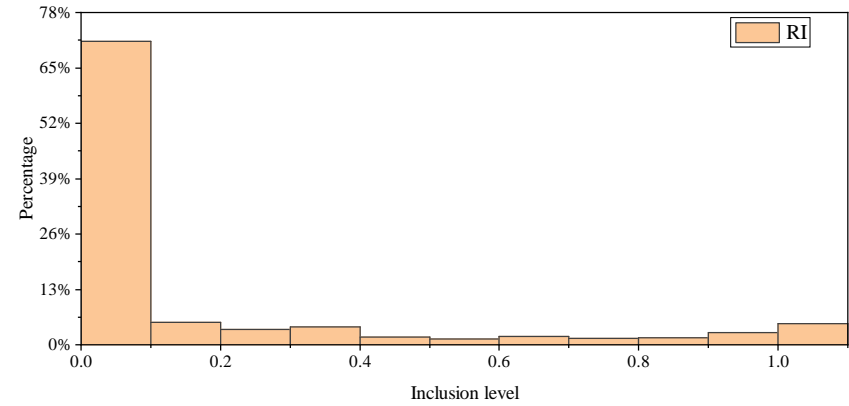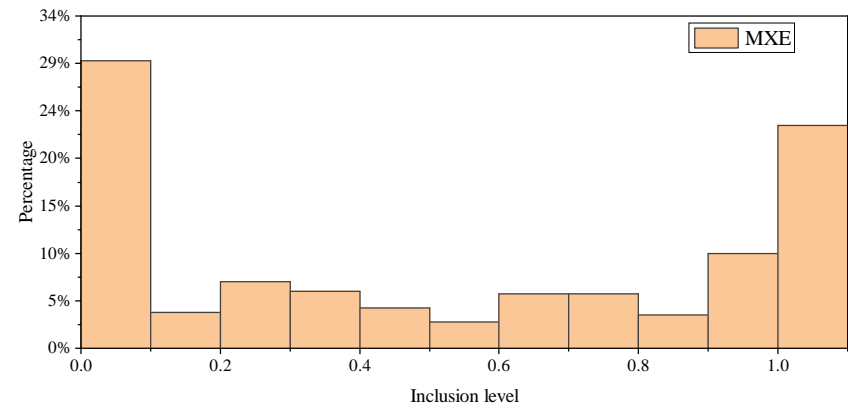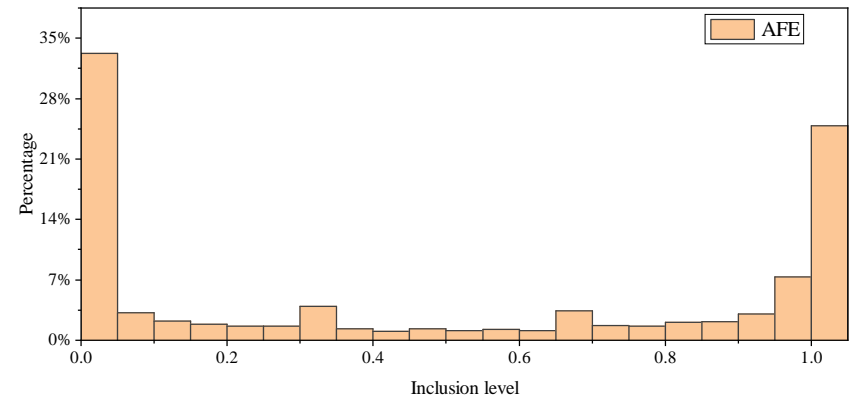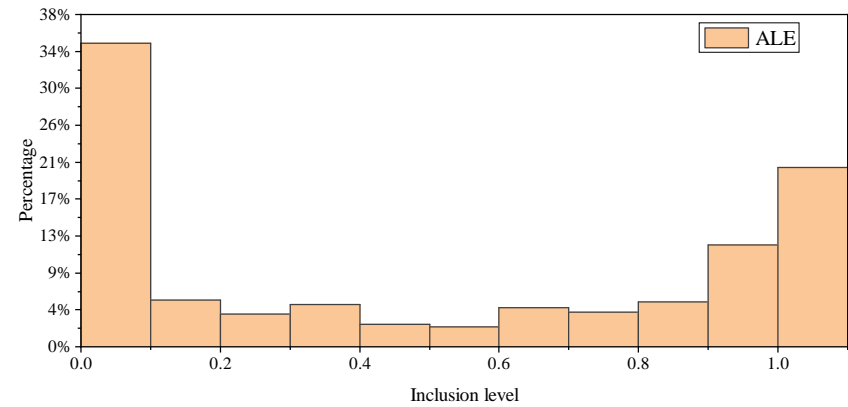

Supplement: Figure S7 — The inclusion ratio distribution of AS events in zygote. [file Data_Sheet_7.PDF]

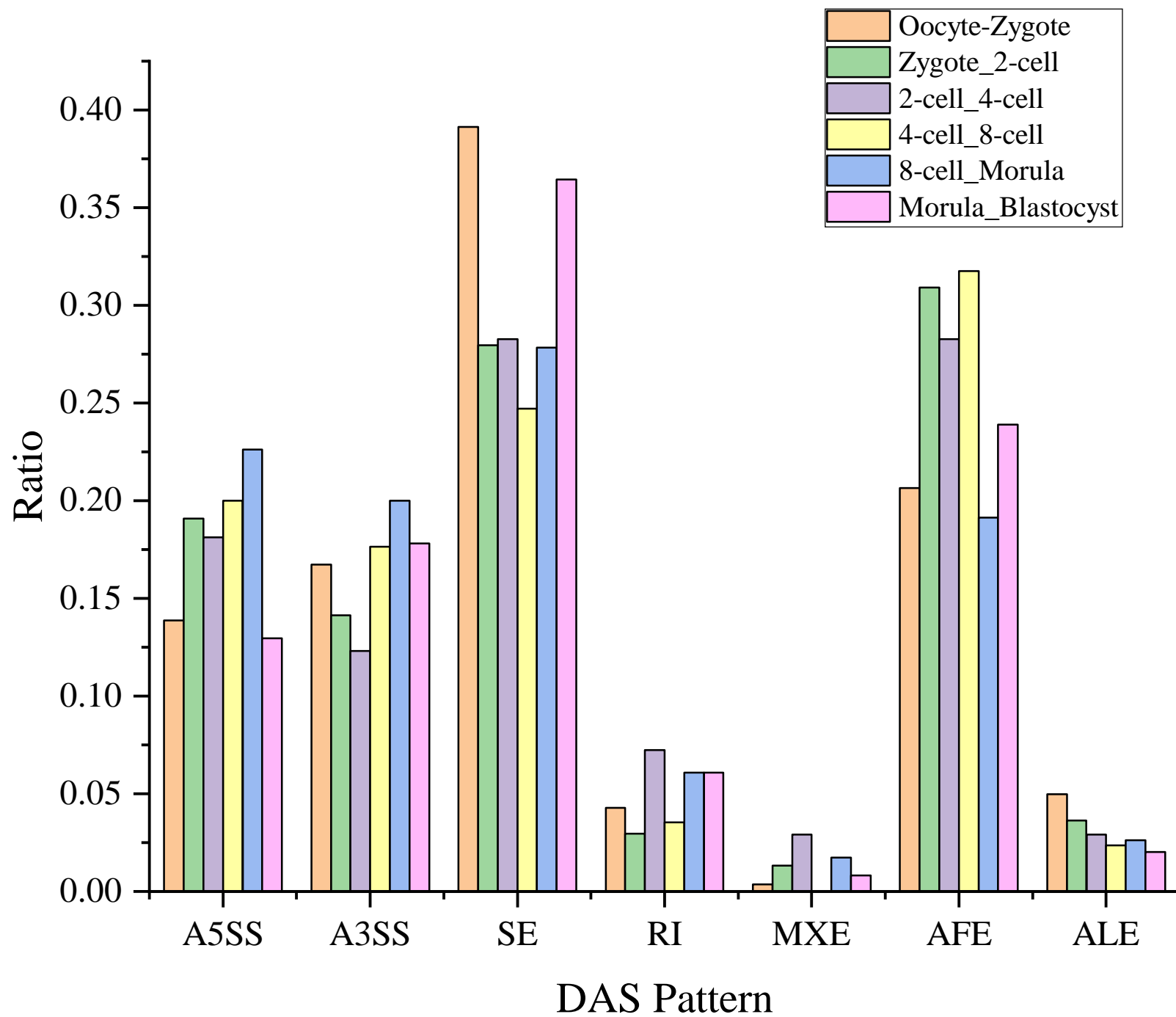

Supplement: Figure S8 — The distribution of DAS pattern between consecutive development stages. [file Data_Sheet_8.PDF]

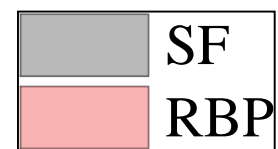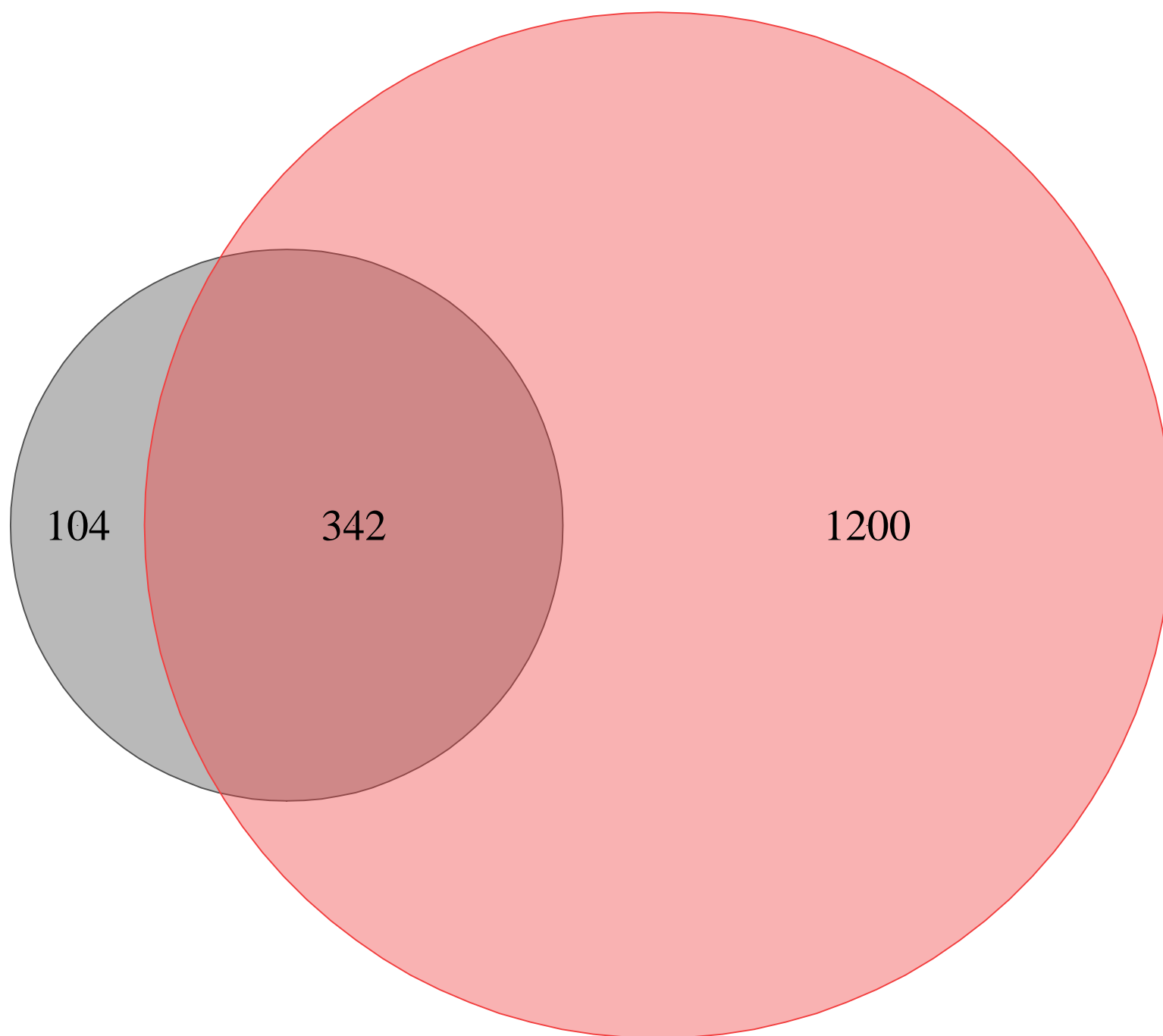

Supplement: Figure S9 — Venn diagram between SFs of mouse and human RBPs. [file Data_Sheet_9.PDF]

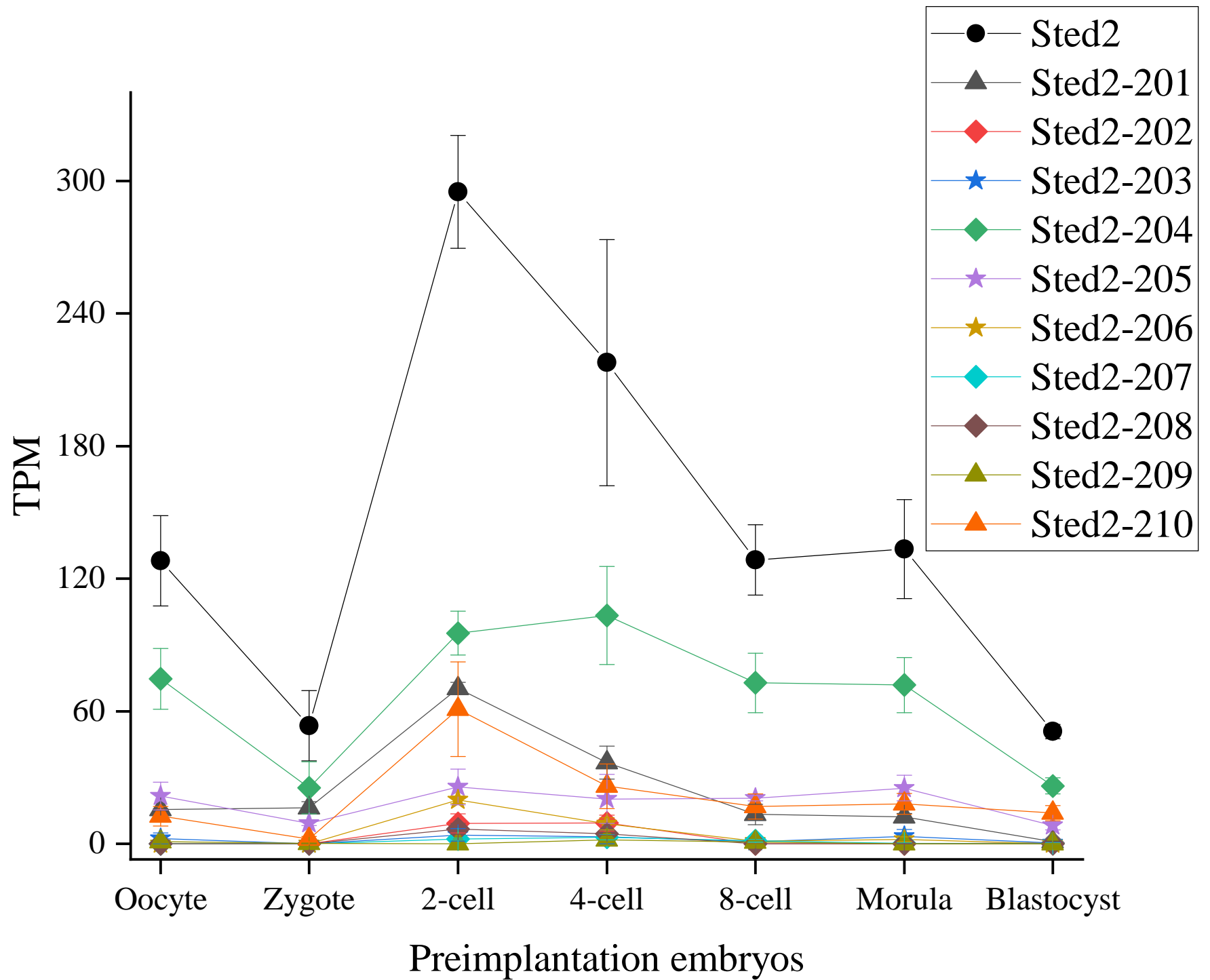

Supplement: Figure S10 — Expression profile of Sted2. The y-axis denotes TPM (Transcripts Per Kilobase Million). The symbol ⊗ denotes switch point. The red line denotes gene expression level and other color lines denote transcript expression level. [file Data_Sheet_10.PDF]

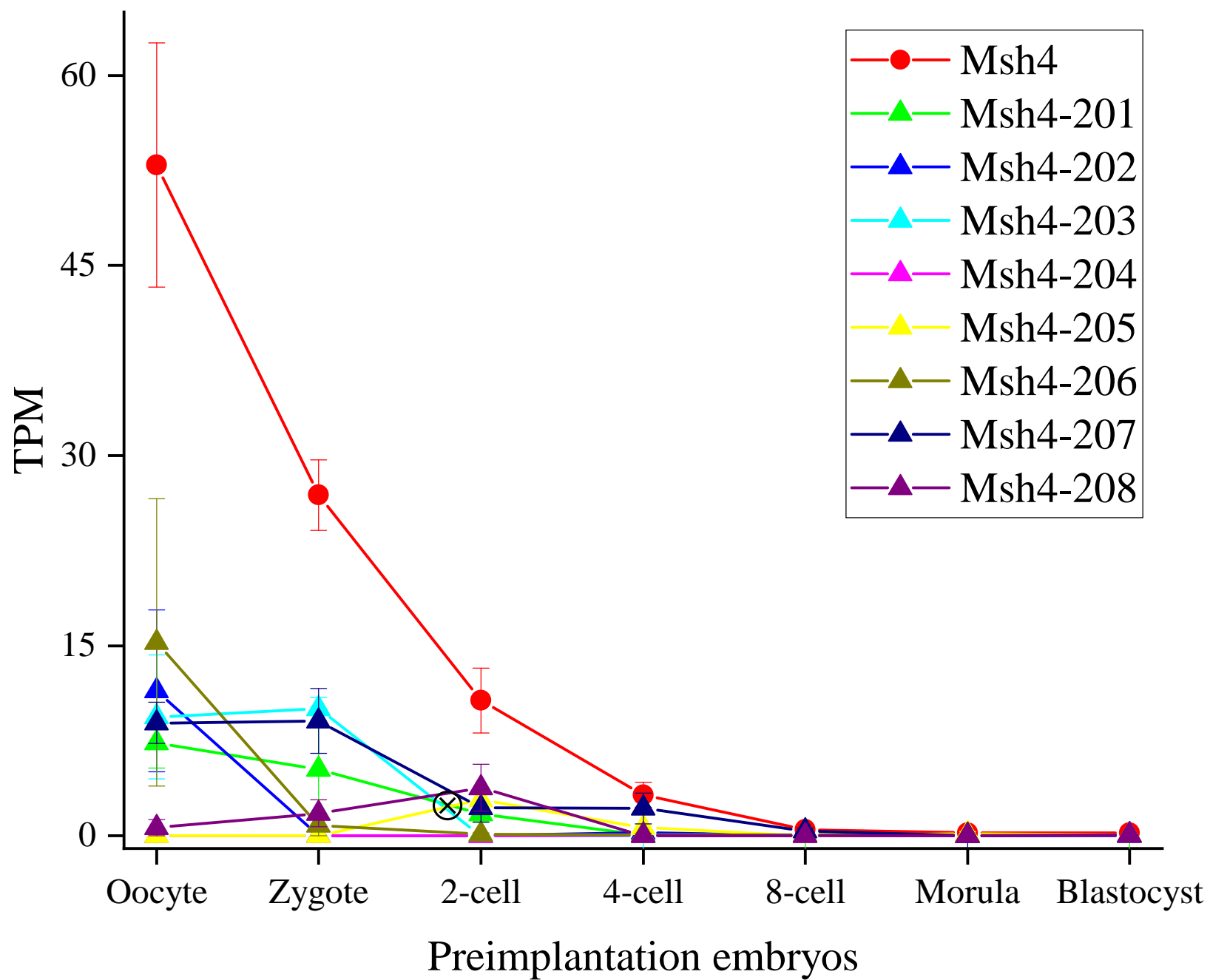

Supplement: Figure S11 — Expression profile of Msh4. The y-axis denotes TPM (Transcripts Per Kilobase Million). The symbol ⊗ denotes switch point. The red line denotes gene expression level and other color lines denote transcript expression level. [file Data_Sheet_11.PDF]

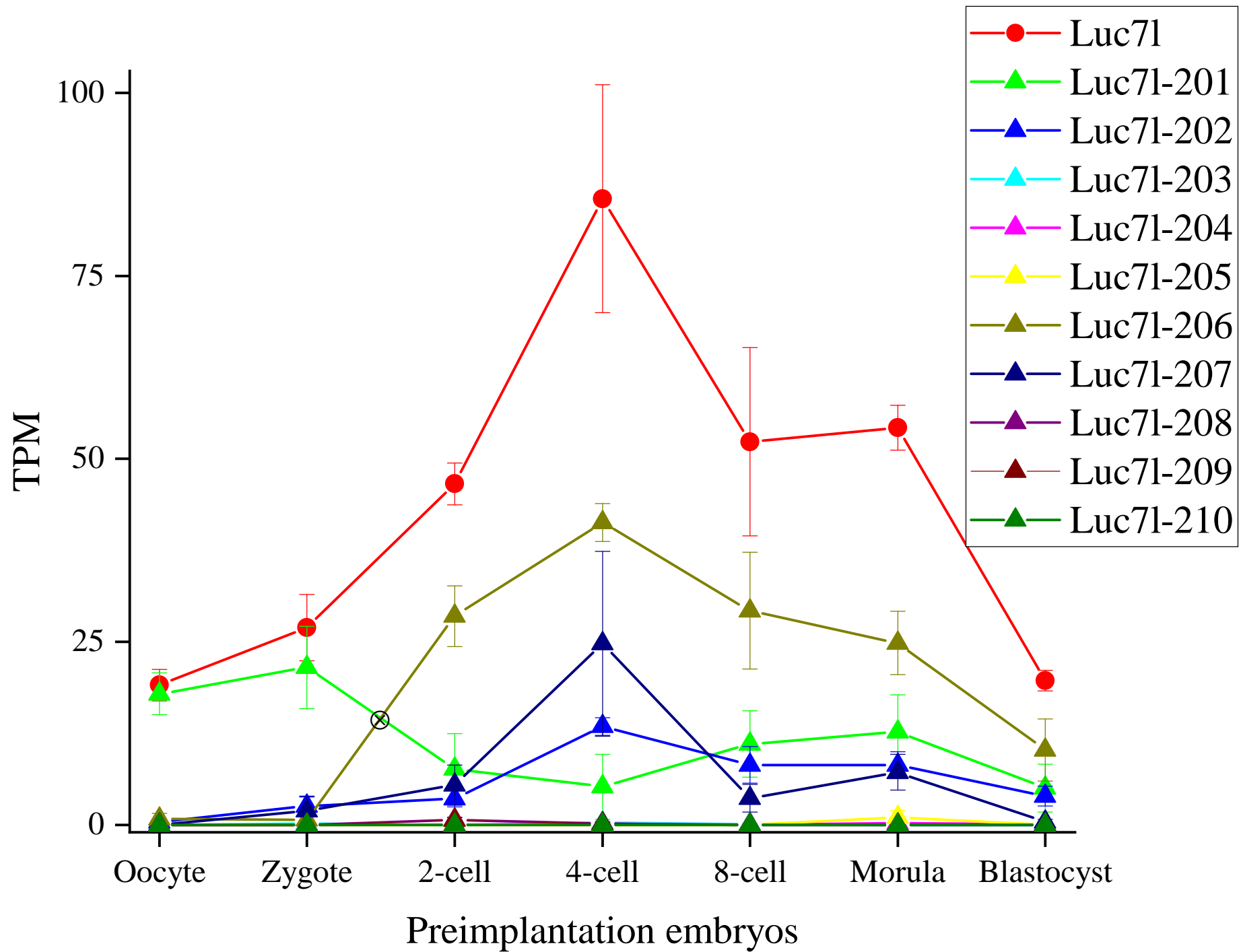

Supplement: Figure S12 — Expression profile of Luc7l. The y-axis denotes TPM (Transcripts Per Kilobase Million). The symbol ⊗ denotes switch point. The red line denotes gene expression level and other color lines denote transcript expression level. [file Data_Sheet_12.PDF]

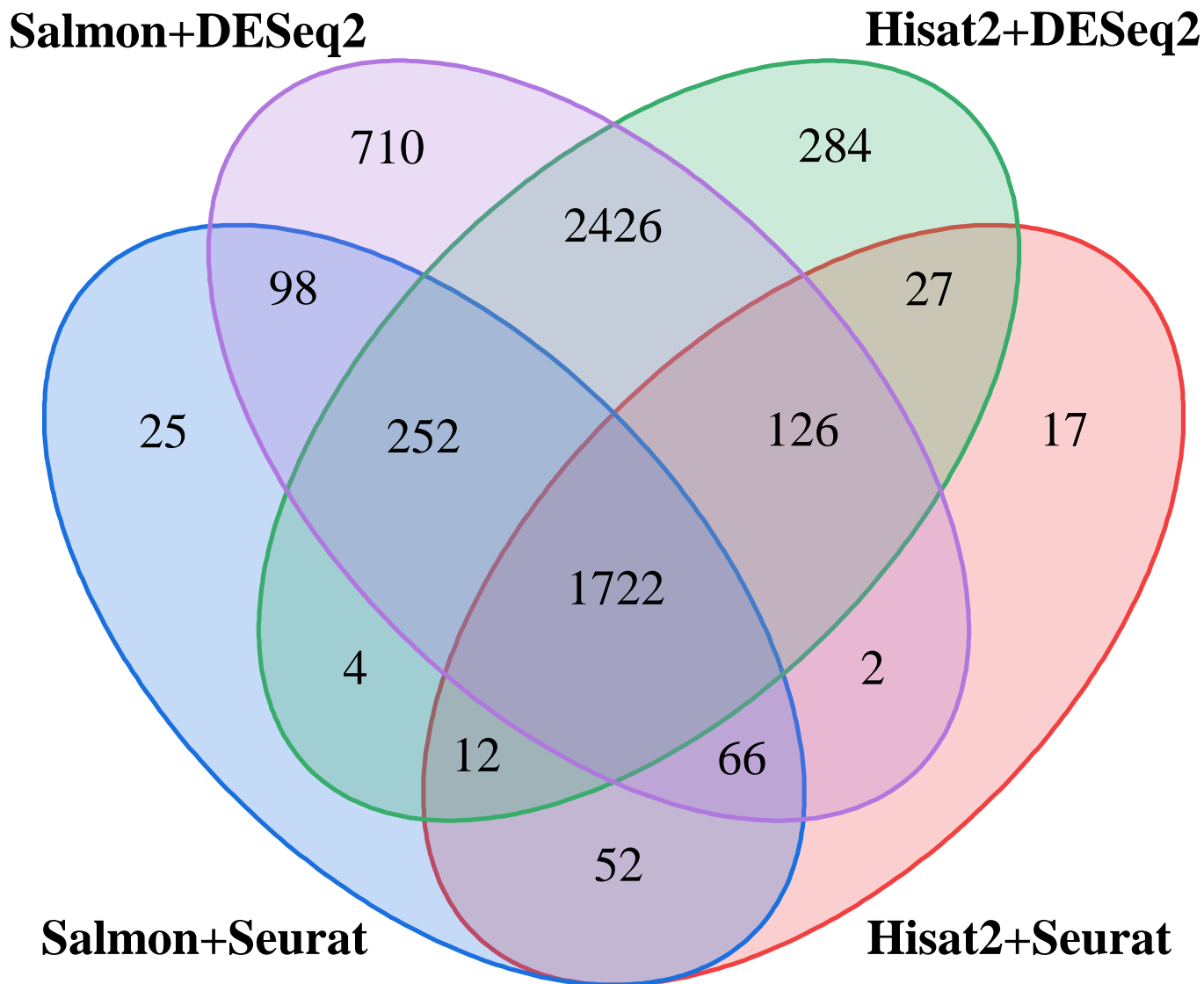

Supplement: Figure S13 — Comparison of different quantifying strategy between 2-cell and zygote. Transcript quantification methods include alignment-free transcript quantification-Salmon and alignment-based transcript quantification-Hisat2. Identifying methods of DE genes include DEseq2 and Seurat. [file Data_Sheet_13.PDF]
