# Supplementary material for: Dynamic Alternative Splicing During Mouse Preimplantation Embryo Development
Source: Front Bioeng Biotechnol. 2020 Feb 7;8:35. doi: 10.3389/fbioe.2020.00035 (PMC7019016; doi:10.3389/fbioe.2020.00035)
Supplement: Figure S4 — The gene expression atlas of significantly down-regulated genes in different preimplantation development stages. The gene expression level was normalized by Seurat (see Materials and Methods). [file Data_Sheet_4.PDF]

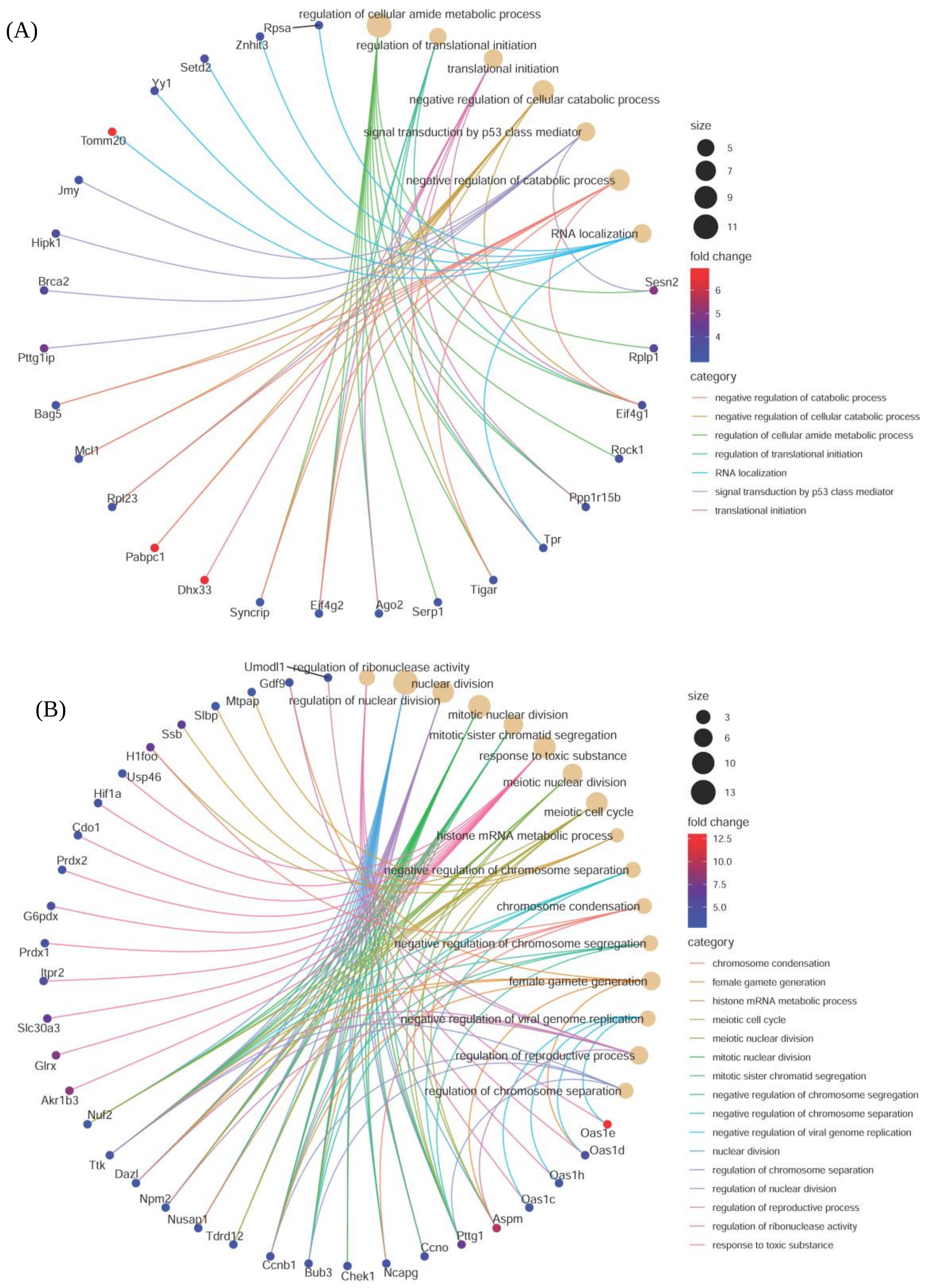

Fig. S2 Functional enrichment analysis of the top DE genes between 2-cell and zygote stages. (A) and (B) represent GO-BP enrichment analysis of most significant DEGs (|Fold Change|>3) between 2-cell and zygotic stages. Circular gene-concept network displayed the linkages of genes and biological concepts.

In order to elucidate the biological function of DE genes more accurately, we also performed GO enrichment analysis of the top DEGs between 2-cell and zygote stages (|Fold Change|>3) (see supplementary text) (See Fig. S2A-B). A total of 7 GO-BP terms corresponding to 134 top up-regulated genes were enriched (Fig. S2A). Obviously, many genes are involved with translational initiation and negative regulation of catabolic process. A total of 16 GO-BP terms corresponding to 152 top down-regulated genes were enriched (Fig. S2B). The majority terms (9/16) belonged to the nuclear division and chromosome separation. It must be emphasized that the response to toxic substance term also significantly enriched. It implied that toxic response is associated with the zygote formation. Apart from these genes that annotated as response to toxic substance and histone mRNA metabolic process, other genes are simultaneously involved with multiple biological processes.
