# Supplementary material for: Dynamic Alternative Splicing During Mouse Preimplantation Embryo Development
Source: Front Bioeng Biotechnol. 2020 Feb 7;8:35. doi: 10.3389/fbioe.2020.00035 (PMC7019016; doi:10.3389/fbioe.2020.00035)
Supplement: Figure S5 — Functional enrichment analysis of the top DE genes between 2-cell and zygote stages. [file Data_Sheet_5.PDF]

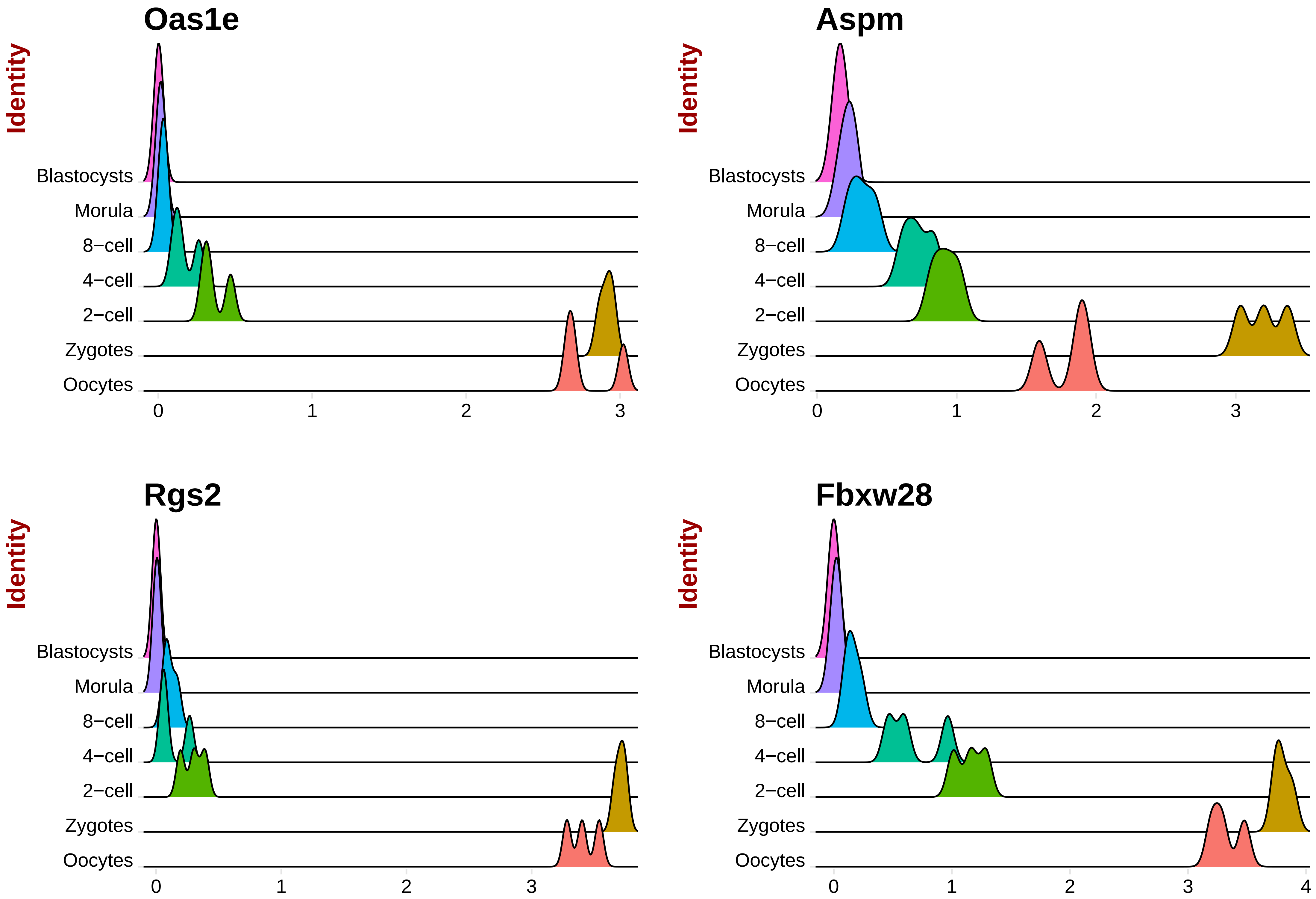

Oas1e
Aspm
Rgs2
Fbxw28
Identity
Blastocysts
Morula
8−cell
4−cell
2−cell
Zygotes
Oocytes
0
1
2
3
4

Oas proteins are produced as latent enzymes, which must bind double-stranded RNA (dsRNA) to form an enzymatically active complex that catalyzes the synthesis of 2′-5′-oligoadenylates (2-5A) from ATP. The 2-5A products bind to latent endoribonuclease RNase L, leading to its dimerization, activation, and degradation of cellular and viral RNA. We observed *Oas* gene family (*Oas1e*, *Oas1d*, *Oas1h*, *Oas1c*) has a biased expression in occyte and zygote stages. The *Aspm* gene is involved in mitotic spindle and cell division (Jiang et al. 2017). Cell division is a critical biological process in occyte and zygote stages. Correspondingly, we also detected the high expression of *Aspm* gene in occyte and zygote stages. *Rgs2* (Regulator of G-protein signalling 2) is involved in G-protein-mediated signaling by negatively regulating the activity of the G-protein α-subunit. It was reported *Rgs2* is expressed in early embryos from the zygote to the blastocyst and reduced levels of *Rgs2* expression lead to abnormal embryonic development in vitro, with embryos arrested at the two- or four-cell stage (Zhang et al. 2015). Our result indicated that *Rgs2* is significantly up-regulated in occyte and zygote stages (See Fig. 3F). The gene expression level of *Fbxw28* which is restricted expressed toward ovary adult is significantly elevated in occyte and zygote stages. We also observed the expression profiles of occyte-specific genes, such as *H1foo*, *Gdf9*, *Bub1b*, *Mos*, etc (Tanaka et al., 2001).

References:

Jiang K, Rezabkova L, Hua S, et al. Microtubule minus-end regulation at spindle poles by an ASPM-katanin complex. Nat. Cell Biol., 2017, 19:480-492.

Zhang Y, You J, Wang X, et al. The DHX33 RNA Helicase Promotes mRNA Translation Initiation. Mol Cell Biol. 2015, 35(17):2918-2931.

Tanaka M, Hennebold JD, Macfarlane J, et al. A mammalian oocyte-specific linker histone gene H1oo: homology with the genes for the oocyte-specific cleavage stage histone (cs-H1) of sea urchin and the B4/H1M histone of the frog. Development, 2001, 128: 655-664.
